# Involvement of redox signalling in tumour cell dormancy and metastasis

**DOI:** 10.1007/s10555-022-10077-9

**Published:** 2023-01-26

**Authors:** Beatriz Puente-Cobacho, Alfonso Varela-López, José L. Quiles, Laura Vera-Ramirez

**Affiliations:** 1 Department of Genomic Medicine, GENYO, Centre for Genomics and Oncology, Pfizer-University of Granada and Andalusian Regional Government, PTS, Granada, Spain; 2 Department of Physiology, Institute of Nutrition and Food Technology “José Mataix Verdú”, Biomedical Research Center, University of Granada, Granada, Spain

**Keywords:** Oxidative stress, Redox signalling, Tumour cell dormancy, Metastasis

## Abstract

Decades of research on oncogene-driven carcinogenesis and gene-expression regulatory networks only started to unveil the complexity of tumour cellular and molecular biology. This knowledge has been successfully implemented in the clinical practice to treat primary tumours. In contrast, much less progress has been made in the development of new therapies against metastasis, which are the main cause of cancer-related deaths. More recently, the role of epigenetic and microenviromental factors has been shown to play a key role in tumour progression. Free radicals are known to communicate the intracellular and extracellular compartments, acting as second messengers and exerting a decisive modulatory effect on tumour cell signalling. Depending on the cellular and molecular context, as well as the intracellular concentration of free radicals and the activation status of the antioxidant system of the cell, the signalling equilibrium can be tilted either towards tumour cell survival and progression or cell death. In this regard, recent advances in tumour cell biology and metastasis indicate that redox signalling is at the base of many cell-intrinsic and microenvironmental mechanisms that control disseminated tumour cell fate and metastasis. In this manuscript, we will review the current knowledge about redox signalling along the different phases of the metastatic cascade, including tumour cell dormancy, making emphasis on metabolism and the establishment of supportive microenvironmental connections, from a redox perspective.

## Introduction

Metastasis is the final step of a complex, multi-stage, and stepwise pathological process, known as cancer progression, which is the main cause of cancer-derived mortality [1]. Through metastasis, tumour cells can disseminate, invade, and colonize distant secondary sites initiating a phase which is incurable from a clinical perspective. Cellular and environmental factors contribute to the process shaping the clinical evolution of cancer patients and highlight the extraordinary complexity of tumour evolution, which is at the base of the inefficacy of current clinical interventions and therapies against metastasis [2].

The heterogeneity of tumour cells regarding their morphology, genetic background, and molecular signalling has been widely described. In addition and related to tumour cell heterogeneity, epigenetic modifications of DNA and/or chromatin in response to different environmental circumstances lead to changes in the selective regulation of gene transcription [3]. The wide variety of cell-intrinsic and cell-extrinsic events, the microenvironmental circumstances faced by a growing tumour mass, together with individual genetic backgrounds, which can either promote or oppose metastasis development, lead to the generation of cell subpopulations characterized by a significant plasticity and increased metastatic potential [3, 4]. However, because of the complexity of tumour growth and progression, it is improbable that isolated genetic alterations are the sole cause of metastasis. Rather, the combination of genetic and epigenetic modifications, together with metabolic adaptations, might be necessary to fuel tumour progression [5].

Metastasis is considered an inefficient process based on the small proportion of cancer cells, known as disseminating tumour cells (DTCs), that colonize an organ which is distant from the anatomical site where the primary tumour originated [6]. Tumour cell dissemination consists of a process known as the invasion-metastasis cascade, which involves different steps. Likewise, it has been proposed [7] that DTCs able to generate a metastatic lesion are distinguished by four features known as the four hallmarks of metastasis: motility and invasion, plasticity, modulation of the local microenvironment of the secondary tissue, and colonization.

Specialized tumour cell subpopulations within the primary tumour mass have been shown to promote tumour cell dissemination based on the expression of a family of metastasis suppressor genes [8]. Although apparently contradictory, the re-expression of such genes in metastasis-promoting tumour cells would block tumour cell dissemination and metastasis growth while stimulating angiogenesis and engaging inflammatory cells. The activation of these processes would promote the development and establishment of premetastatic niches through the communication of cells from the primary tumour with other sites of the body [9]. Indeed, it is known that metastasis development is initiated long before any metastatic tumour mass is detectable, as initially described by Drs. Kaplan and Lyden [9–11], due to the establishment of supportive metastatic environments induced by the primary tumour though the secretion of soluble factors and the recruitment of hematopoietic and mesenchymal stem cell populations that mobilize and condition the secondary site.

Likewise, tumour cell invasion requires alterations in the surrounding environment, as well as modifications in cell morphology and phenotype. During this step, there are three processes that have been considered as dynamically regulated: adhesion, reorganization of the extracellular matrix (ECM) and motility through the contractility of the cytoskeleton.

Adhesion is mediated by integrins and transmembrane glycoproteins. These surface molecules are considered the main cellular adhesion receptors at the interface with the ECM. Furthermore, integrins are associated with almost every step of the metastasis cascade through their diverse functions as signalling molecules, mechanotransducers, and crucial components of the cell migration machinery [12]. These molecules are cell surface heterodimers that link the actin cytoskeleton to the cellular membrane and mediate cell-ECM interactions. The strength of the cellular adhesion to the ECM might be regulated through cell-intrinsic signalling pathways, whereas cellular phenotype can be modified through changes in cellular adhesion, highlighting the bidirectional nature of this interaction [12, 13].

On the other hand, ECM remodelling occurs through the release of degradative enzymes by tumour cells or cells associated to the tumour. It has been described that these enzymes can act alone or together, through interactions that modulate their catalytic activity. Serine proteinases, cysteine proteinases, aspartyl proteinases, and matrix metalloproteinases (MMPs) are proteases well known to play a key role in tumour invasion [14]. However, the function of some of these proteolytic enzymes, specifically MMPs, is not only the physically demolition of ECM barriers but also the modulation of several other cellular processes (such as cell growth, differentiation, apoptosis, angiogenesis, chemotaxis, and migration) through their substrates and cleavage products [15, 16].

Finally, motility during tumour cell invasion has been distinctly described depending on whether it involves a single cell or a group of cells moving in concert through a mechanism known as collective migration [17]. Cell migration is classified as mesenchymal or amoeboid, when referring to single cell migration, and collective when migration involves a coordinated group of cells. These three modes or types of cellular migration are interconvertible depending on the modulation of cytoskeletal structure [18] and the relative levels of adhesion, cellular, and nuclear deformability [19]. At the dynamical front edge of amoeboid migration, two types of cellular protrusions have been distinguished: amoeboid blebby and ameboid filopodial/pseudopodal. Amoeboid migration is also characterized by ability of the cells to deform their body, maintaining the tissue architecture and weakly adhering to its ECM [20]. Conversely, mesenchymal migration showed a stronger adhesion and capability of tissue realignment and remodelling through deposition of ECM and cytokines [21]. Cells undergoing mesenchymal migration are characterized by an elongated shape, decreased cell–cell interactions, and increased motility. Tumour cells are known to achieve this characteristic morphology through a process known as epithelial-to-mesenchymal transition (EMT). EMT is characterized by a decrease in the cellular expression of epithelial-specific Cadherin-1 (CDH1) and the increase of Cadherin-2 and/or -3 (CDH2 and CDH3, respectively) enabling more motility and individual migration in the cells [22]. EMT will be further discussed in the context of redox signalling in metastasis, as part of specific sections in this review. Regarding to collective migration, three additional subtypes of collective movement have been described, which are characterized by an increasing degree of cell–cell adhesion: neuronal, epithelial (sheet/strand or ductal/glandular), and endothelial (vascular) collective migration. The morphological and molecular mechanisms regulating collective migration have been recently reviewed elsewhere and the readers are referred to these articles for more information [23, 24].

Once local invasion is completed, cells must intravasate the circulatory system becoming circulating tumour cells (CTCs). To the succeed of this step, the ECM and the basement membrane must be partially degraded, so tumour cells can push between endothelial cells extending filopodia into the lumen, preserving the integrity of the endothelial barrier [7, 25]. Although the metastatic process is highly inefficient, early steps in the metastatic cascade are achieved by tumour cells more frequently than later steps. For example, the number of tumour cells shed into the bloodstream is significantly higher than the number of tumour cells that eventually will give raise to an active metastatic lesion [26, 27]. During their journey through the bloodstream, tumour cells are in contact with leukocytes, lymphocytes, and other immune components. Tumour cells have been observed to evade immune system by downregulating the expression of antigens, producing factors that help tumour cells to be recognized as “normal,” or directly killing immune cells. Despite the high degree of adaptability to a hard microenvironment, as it is the bloodstream, a significant proportion of the shed CTCs succumb to the exposure to hydrostatic pressures and hemodynamic shear forces [28]. Even when CTCs are characterized for being deformable, cell origin and biophysical parameters may determine if the cell will survive or will be broken by shear. Interestingly, to protect cells from shear forces and immune attacks, cancer cells can be transported as an embolus or a CTC cluster [29].

After surviving shear forces, tumour cells adhere in a selective manner to the endothelium. Endothelial cells found in each tissue express characteristic markers or a combination of them that can be recognized by tumour cells, promoting selective infiltration [30–32]. Apparently, tumour cells adhere in a more efficient manner at anatomical locations where inflammation takes place. After recognition and adherence to the endothelium, cancer cells migrate through it and encounter the basement membrane. At the basement membrane, cancer cells produce proteinases that lead to its deformation and squeeze between endothelial cells and through holes in the matrix [7, 33]. Extravasation was previously thought to be a limiting step of the metastatic cascade, but even its requirement for the completion of the metastatic process has been discussed, as it has been observed that tumour cells can form pulmonary metastases through the attachment to the lung endothelium and intravascular growth [34]. Next, tumour cells start their interaction with the premetastatic niche, which promotes the proliferation and colonization of that secondary site. At this point, colonization depends on the ability of DTCs to interpret and adapt to the new tissue microenvironment, which will determine tumour cell fate. On one hand, tumour cells can remodel the microenvironment to allow continued growth, leading to the development of a clinically detectable metastatic lesion [35, 36]. Interestingly, the requirements for colonization of secondary tissues are similar to those of the primary tumour, such as be provided of enough oxygen and nutrients [37, 38], but in the secondary sites, additional players determine DTCs’ fate. In this regard, immune cells have been shown to play a role in the establishment of the premetastatic niche and tumour cell colonization. Neutrophils form the so-called neutrophil extracellular traps (NETs) by the extracellular deposition of DNA, which promote DTC colonization and growth [39]. Conversely, DTCs require to evade immune surveillance and clearance to successfully colonize secondary sites and grow into a metastatic lesion. Malladi et al. [40] showed that DTCs self-impose a slow-cycling state through the autocrine inhibition of Wnt signalling and downregulation of natural killer (NK) lymphocyte ligands, which impeded NK-mediated recognition and cytolysis and promoted immune evasion. The “slow-cycling state” described by the authors alludes to a stage of metastasis known as tumour dormancy.

As described above, metastasis is a stepwise process; the kinetics of which are highly variable across the different tumour malignancies and within different subtypes of neoplasms originated at the same anatomical primary location [7]. The highly heterogeneous rates of recurrence in breast cancer, depending on the estrogen receptor (ESR) status of the tumour, exemplify this phenomenon. Breast cancer patients diagnosed with ESR-negative tumours have a higher risk of recurrence during the first 5 years after diagnosis, as compared to patients diagnosed with ESR-positive tumours. On the contrary, patients diagnosed with ESR-positive tumours show a higher steady rate of recurrence that expands from 5 to 20 years from diagnosis [41, 42]. This highly variable and potentially long period from tumour diagnosis to the detection of a metastatic lesion may result from DTCs. DTCs are thought to prevail in a dormant state until the appropriate stimuli trigger a switch that promotes colonization of the microenvironment and proliferation in the metastatic site, resuming tumour progression and the emergence of a clinically active metastatic lesion [6]. Different concepts have been coined in relation to tumour dormancy. Tumour dormancy can be the result of a proliferative arrest at the G0 phase of the cell cycle (tumour cell dormancy), or the consequence of a balance between proliferation and apoptosis of cancer cells (tumour mass dormancy) [43, 44]. On one side, data from different studies have associated apoptosis in tumour mass dormancy with the inexistence of a proper vascularity providing sufficient nutrients and oxygen, known as angiogenic dormancy [45, 46]. On the other side, tumour mass dormancy has also been linked to the response of the immune system, probably due to the coordinated activity of cytotoxic CD8 + T cells, memory T cells, and humoral response, which is called immune system-induced dormancy [43]. Despite the potential involvement of these different and presumably non-exclusive modes of tumour dormancy along the metastatic cascade, tumour cell dormancy has been best characterized, both at the molecular and clinical levels, in the context of metastasis.

As mentioned before, microenvironment has been shown to play a key role in the establishment and induction of tumour cell dormancy. Early seminal studies have demonstrated that DTCs undergo growth arrest and enter dormancy when they are not able to achieve appropriate interactions with the ECM. Supporting this idea, Aguirre-Ghiso et al. [47] described that the *in vivo* downregulation of urokinase plasminogen activator receptor (uPAR) induces tumour dormancy by inhibiting the interaction of the uPA/uPAR proteins with the α5β1 integrin. The downregulation of this interaction decreased tumour cell adhesion to fibronectin and reduced the activation of mitogen-activated protein kinase (MAPK)/extracellular signal-regulated kinase (ERK) pathway. An additional study reported that the uPA/uPAR interaction activate the generation of insoluble fibronectin fibrils inhibiting p38 MAPK activity. Furthermore, the authors also concluded that depending on p38 MAPK/ERK activity ratio Hep3 cells, a model of human head and neck squamous cell carcinoma (HNSCC) showed a distinct proliferative status. Hep3 cells showed growth arrest when the ERK/p38 ratio was high due to p38 phosphorylation and activation [48]. Interestingly, downregulation of uPAR in Hep3 cells inhibited focal adhesion kinase (FADK 1) phosphorylation and downstream proto-oncogene tyrosine-protein kinase Src (Src) activation leading to cellular dormancy *in vivo* [49].

In line with these findings, MDA-MB-231 breast cancer cells infiltrated into the bone marrow have been shown to upregulate the expression of Src kinases and upregulate the phosphoinositide 3-kinase (PI3K)/protein kinase B (PKB, also named AKT) pathway, as a mechanism of survival in response to stromal cell-derived factor 1 (SDF-1)/C-X-C chemokine receptor type 4 (CXCR-4) and tumour necrosis factor ligand superfamily member 10 (TNFSF10, also named TRAIL), which are highly expressed in the bone microenvironment [50]. Barkan et al. found that the dormant-to-proliferative switch was dependent on the engagement of integrin β1 (ITGB1) and downstream signalling via activation of FADK 1, Src, ERK, and myosin light-chain kinase (MLCK) in breast cancer D2.0R cells. They demonstrated that ITGB1-mediated phosphorylation of myosin light chain by MLCK is necessary for the development of actin stress fibers and proliferative growth. When either ITGB1 or MLCK were inhibited, the dormant-to-proliferative switch was prevented and a significant reduction of the metastatic burden was observed *in vivo* [51]. In addition, the authors showed that the dormant-to-proliferative switch was driven by cytoskeletal reorganization in otherwise dormant D2.0R cells due to the enrichment of the metastatic niche with type I collagen [52]. Lastly, the team showed that combination treatment eliciting the simultaneous inhibition of Src and ERK1/2 could induce apoptosis in dormant breast cancer cells [53]. In addition, a recent study revealed that D-HEp3 (dormant HNSCC) DTCs reorganize the ECM through the secretion of type III collagen via DDR1-mediated STAT1 signalling to maintain tumour cell dormancy. Conversely, the elimination of collagen III re-established the proliferative behavior of cancer cells and changes in the structure and quantity of collagen during the dormant-to-proliferative transition were observable [54].

Other components of the microenvironment, such as the vascular system, have also been studied in the context of tumour cell dormancy. The endothelium has been shown to promote breast tumour cell dormancy via the expression and release of thrombospondin-1 (TSP-1). Conversely, the authors found that DTC proliferation was activated by endothelial tip cells in the neovasculature through secretion of transforming growth factor-beta (TGFβ) and periostin [55, 56]. Finally, a recent study showed that the interaction of breast DTCs with resident epithelial cells of the secondary microenvironment significantly impacted tumour cell dormancy. Montagner et al. [57] showed that dormant breast DTCs in the lung interacted with alveolar type 1 epithelial cells leading to the formation of fibronectin fibrils and driving integrin-mediated pro-survival signals. Mechanistically, the interaction was mediated by the secreted frizzled-related protein 2 (FRP-2), whose expression inhibition reduced the number of dormant breast DTCs.

While environmental factors have been studied and thoroughly discussed in the context of tumour dormancy induction and maintenance, only few studies have addressed the intrinsic molecular mechanisms which allow dormant DTCs to survive in the metastatic niche for extended periods. In this regard, autophagy, which is an evolutionary conserved mechanism of cell survival opposing metabolic stress, has been shown to promote dormant DTCs survival [58]. Interestingly, this and other studies that will be discussed later in this review highlight the key role of redox signalling and the intracellular redox balance in tumour cell dormancy and metastasis. Herein, we will review the current experimental evidence showing the determining role of redox biology and redox signalling along the metastatic cascade. In addition, we will discuss the potential therapeutic implications of such knowledge in the design and implementation of new and improved antineoplastic therapies.

## Redox signalling in tumour progression

ROS are produced intracellularly as a by-product, by mitochondria and other cellular elements, and exogenously by pollutants, tobacco smoke, drugs, xenobiotics, and radiation. Cancer cells exhibit persistently high levels of ROS because of genetic, metabolic, and microenvironment-associated instability [59–61]. Therefore, cancer cells are chronically exposed to sublethal levels oxidative stress which are known to modulate cell signalling and fate. At this point, it is worth to review the current concept of oxidative stress, which has evolved since it was first introduced by the scientific literature. Oxidative stress is considered a cellular state in which living cells are exposed to highly reactive oxidizing molecules. Consequently, the pro-oxidant/antioxidant balance of the cell is disrupted in favor of pro-oxidant processes leading to a wide variety of cellular responses, ranging from the modulation of signalling networks to apoptosis due to persistent and/or severe oxidative damage [62–64].

Accumulated evidence has suggested that highly metastatic cancer cells contain high levels of ROS and that intracellular redox state governs crucial steps for the metastatic process promoting cell invasion and metastatic spread. Early experiments showed that treatment of carcinoma cells with H_2_O_2_ prior to intravenous injection into mice enhanced metastasis [65]. Additionally, subpopulations of the low- or non-motile breast cancer cell line MCF-7 that possess higher levels of endogenous ROS, as compared to the parental cell line, showed increased motility. In addition, orthotopic breast tumours generated with these “high endogenous ROS” cell subpopulations metastasized to the lung, liver, and spleen while the orthotopic tumours generated using the parental MCF-7 line did not [66]. The role of ROS in metastasis is also supported by the fact that ROS attenuation by antioxidants suppressed hypoxia-induced metastasis of human pancreatic cancer cells in a xenograft nude mouse model [67].

Mechanistically, ROS have been found to increase the expression and/or activate MMPs, adhesion molecules [68], epidermal growth factor (EGF) [69], epidermal growth factor receptor (EGFR) [70], and vascular endothelial growth factor (VEGF) [71] whose upregulation and activity is known to be crucial along the metastatic cascade. Several genes relevant to EMT, including CDH1, integrins, and MMPs, have shown to be directly or indirectly regulated by intracellular ROS levels [71]. Interestingly, the dismutation of mitochondrially generated superoxide to H_2_O_2_ is considered an important step in oxidative stress-mediated expression of MMP genes [72]. In this line, the treatment of SCp2 mouse mammary epithelial cells with the ROS scavenger N-acetyl-L-cysteine (NAC) abolished EMT though the inhibition of the expression of MMP-3, a stromal protease that is upregulated in mammary tumours [73]. Additionally, to modulate expression of MMP genes, ROS can lead to the direct activation of MMPs through reaction with thiol groups in their catalytic domain [74]. Interestingly, ROS regulate not only the expression and activity of MMPs, but also the inactivation of their inhibitors, such as the metalloproteinase inhibitor (TIMP) [75, 76]. Increased MMP activity has also been associated with angiogenesis, increased tumour cell invasion, and blood vessel penetration [77–80]. A role for ROS in angiogenesis though MMPs increase has been evidenced. ROS-induced secretion of MMP-1 from tumour cells promoted vessel growth within the tumour microenvironment [81]. Moreover, a transient expression of MMP-1, MMP-2, and MMP-9 correlated with an increase in ROS during formation of capillary-like structures, implicating that MMP-mediated angiogenesis also occurs through upregulation of ROS [82]. On the other hand, cancer cells have been shown to purposefully restrain pyruvate from entry into mitochondrial oxidative metabolism, given that the ROS produced as by-products of mitochondrial respiration exhibit anti-metastasis activity [83].

Resistance to anoikis and independence from cell attachment signals promote tumour cell survival through increased generation of intracellular ROS. It has been suggested that an increase in oxidative stress mimics autocrine/adhesive signals, which in non-tumour cells is mediated by growth factor- and integrin-mediated signalling pathways [70, 84, 85]. The activation of these signalling pathways would contribute to increase the threshold for anoikis induction in cancer cells, elevating their disseminating and metastatic potentials. Thus, metastatic cancer cells gain increased anoikis resistance and survival advantage through increased intracellular ROS generation and ROS-mediated signalling.

ROS have also been reported to participate in the regulation of mesenchymal-like tumour cell movement that likely involve, besides gene regulation, a direct modification of cytoskeleton dynamics through actin glutathionylation [85, 86]. In general, ROS appear to promote an “explorative” behavior whereby membrane protrusions and fast-turnover focal contacts with ECM prevail over stable focal adhesions and cell contractility. These characteristics are typically observed in invadopodia, the actin cytoskeleton-based structures that tumour cells use to invade. NADPH oxidases (NOX) have been found, concentrated, and activated, at the invadopodia of several types of malignant cells [87–89]. NOX1-mediated ROS generation has been shown to be necessary for the formation of invadopodia [88], where also MMP activity is concentrated. Accordingly, invadopodia formation is impaired in the absence of NOX-derived ROS [88]. ROS generated by NOX have been show to activate the cofilin pathway and thus contribute to increased cell migration [90, 91].

ROS may also promote tumour cell metastasis by increasing vascular permeability [77] and triggering vasodilation through activation of the enzyme heme oxygenase 1 (HO-1), given that HO-1 is able to induce the formation of nitric oxide [92]. The sources of ROS and their importance differ along the different steps of the metastatic process. Elevated ROS levels resulting from mutations in mitochondrial DNA have also been shown to promote metastasis [70, 93]. Cell detachment during metastasis upregulates pyruvate dehydrogenase kinase 4 (PDK4) which inhibits pyruvate dehydrogenase complex (PDHc) and decreases the flux of glucose carbon into the tricarboxylic acid (TCA) cycle [94]. On the other hand, cell adhesion and migration are dependent on integrin binding to ECM and these are able to elevate oxidant levels mainly by increasing prostaglandin G/H synthase 2 (PGHS-2, also named COX-2) [68] but also through polyunsaturated fatty acid 5-lipoxygenase (5-LO) and even mitochondria [68, 84]. In this context, an increase in mitochondrial ROS was linked to a first cellular contact with the ECM and increases in cytosolic ROS were shown to contribute to cytoskeleton remodelling and actin stress fiber formation during a later phase of the process [84, 95]. In turn, these increases in ROS can trigger oxidative stress leading to oxidative damage to DNA and genomic instability. Despite NOXs have been clearly involved in invasion-related redox signalling, mitochondria may also contribute as sources of oxidant species in malignant growth [96–99].

On the other hand, the effects of ROS are not specific to cancer cells and may result in the destruction of normal cells and tissues as well. Changes in the tumour microenvironment can bring about invasion and adhesion processes. Oxidative stress initiated in tumour cells is transferred to cancer-associated fibroblasts laterally and vectorially via H_2_O_2_. Excess of stromal ROS production has been shown to drive the onset of antioxidant defense in adjacent cancer cells, protecting them from apoptosis. Moreover, ROS can also act as players of immune regulation in cancer development. In particular, ROS are likely to participate as immunosuppressive agents [100–102] in the tumour microenvironment facilitating tumour invasion, metastasis, and resistance. Most ROS-sensitive pathways transduce cytoplasmic signals to the nucleus, where they influence the activity of transcription factors that control the expression of a wide array of genes. In this regard, to prevent excessive intracellular ROS, cancer cells respond to oxidative stress by inducing the transcription of antioxidant enzymes, highlighting the relevance of an in-depth knowledge of these pathways for use in elaborating therapies that alter ROS levels.

Finally, antioxidant defense systems are expected to be also key signalling elements since they also modulate redox state. High levels of ROS are usually compensated by increased antioxidant capacity of the cancer cells. Due to the persistent high ROS microenvironment and increased intracellular ROS levels, cancer cells adopt efficient mechanisms of ROS detoxification. Consequently, they show high dependency on antioxidant systems for their survival. The cell’s defense against ROS includes antioxidant enzymes that detoxify ROS and prevent their intracellular accumulation at high concentrations [103]. Evidence suggests that cancer progression involves numerous alterations in specific metabolic pathways involved in synthesis of proteins, lipids, and nucleotides. Besides, there is an increase in the generation of reductive equivalents, such as NADPH or GSH, and redox cofactors, such as NADH and flavin adenine dinucleotide (FADH2). There is a reciprocal crosstalk between metabolism and redox balance in cancer cells, with a particular emphasis on the role of glycolysis, glutaminolysis, fatty acid oxidation, one-carbon metabolism, and the pentose phosphate pathway [104, 105]. Cell detachment has been shown to increase the expression of manganese superoxide dismutase (MnSOD), the main mitochondrial antioxidant enzyme, to detoxify mitochondrial ROS resulting from detachment [106]. Moreover, it has been found that cells depleted of MnSOD were hypersensitive to matrix detachment [106]. Therefore, through the activation of the antioxidant systems, cancer cells gain increased anoikis resistance and a survival advantage for the completion of subsequent steps of metastasis. However, metastatic breast cancer and highly invasive pancreatic cancer cells show lower levels and activity of the antioxidant enzyme MnSOD [107–109]. Other redox proteins with redundant functions, such as thioredoxin and peroxiredoxin, may contribute to survive the raise in oxidative stress caused by anoikis. Indeed, it has been shown that reverse (basolateral-to-apical) transendothelial migration of human melanoma cells is induced by H_2_O_2_ and can be blocked by thioredoxin [110].

Therefore, redox state has a profound impact on intracellular cell signalling [111]. Not surprisingly, it is considered that ROS function as second messengers to regulate multiple metastasis-related signalling pathways by interacting with different proteins [112]. From a biochemical standpoint, the oxidation of redox-sensitive cysteine and/or tyrosine residues located within or around the active site of many enzymes generates intra- and inter-protein bridges that affect their function [113, 114]. These modifications generate a wide array of cellular responses [115]. The possible mechanism involved in promoting targeted protein oxidation by H_2_O_2_ may involve the ability of ROS-scavenging enzymes, such as glutathione peroxidase (GPx), to sense and transduce the H_2_O_2_ signal, which is classified as a redox-relay mechanism. Another mechanism proposed is the so-called floodgate model in which oxidation causes inactivation of the ROS-scavenging enzymes by hyperoxidation or phosphorylation of key aminoacids, causing localized increases in H_2_O_2_ leading to protein oxidation and loss of function [116].

ROS have been shown to regulate numerous signalling pathways (e.g., the MAPK and PI3K/AKT pathways) and activities of key transcriptional factors (e.g., hypoxia-inducible factor (HIF) and zinc finger protein SNAI1 (SNAI1)) to enhance cancer cell migration and invasion. Notwithstanding, ROS are also associated with epigenetic changes in genes. It is established that many transcription factors, including activator protein 1 (AP-1), hypoxia-inducible factor 1-alpha (HIF-1α), heat shock transcription factor 1 (HSF-1), nuclear factor kappa-light-chain-enhancer of activated B cells (NF-κB), nuclear factor erythroid 2-related factor 2 (Nrf-2), and tumour protein p53 (TP53) are activated by ROS and regulate the redox status of cells [117]. The extent to which individual members of the abovementioned network of antioxidant transcription factors are differentially activated by oxidative stress is uncertain, although it is improbable that all are activated simultaneously. Rather, different transcription factors likely respond to distinct threshold levels of ROS/reactive nitrogen species (RNS), in a concentration- and/or time-dependent manner that is probably attuned to the coexistence of metabolic stress, proteotoxic stress, hypoxia, inflammation, or DNA damage. While these transcription factors have all been experimentally shown to be involved in carcinogenesis, more recent studies show that they also contribute to redox status and are implicated in tumour progression [118].

From the extensive collection of experimental data summarized in this section, it is possible to conclude that the intracellular redox status of cancer cells has a profound impact on metastasis and tumour progression. In the coming sub-sections of this review, we will review and analyze the scientific evidence investigating the role of redox signalling along the different stages of metastasis.

## Redox signalling during tumour cell invasion and dissemination

### EMT

**Fig. 1:**
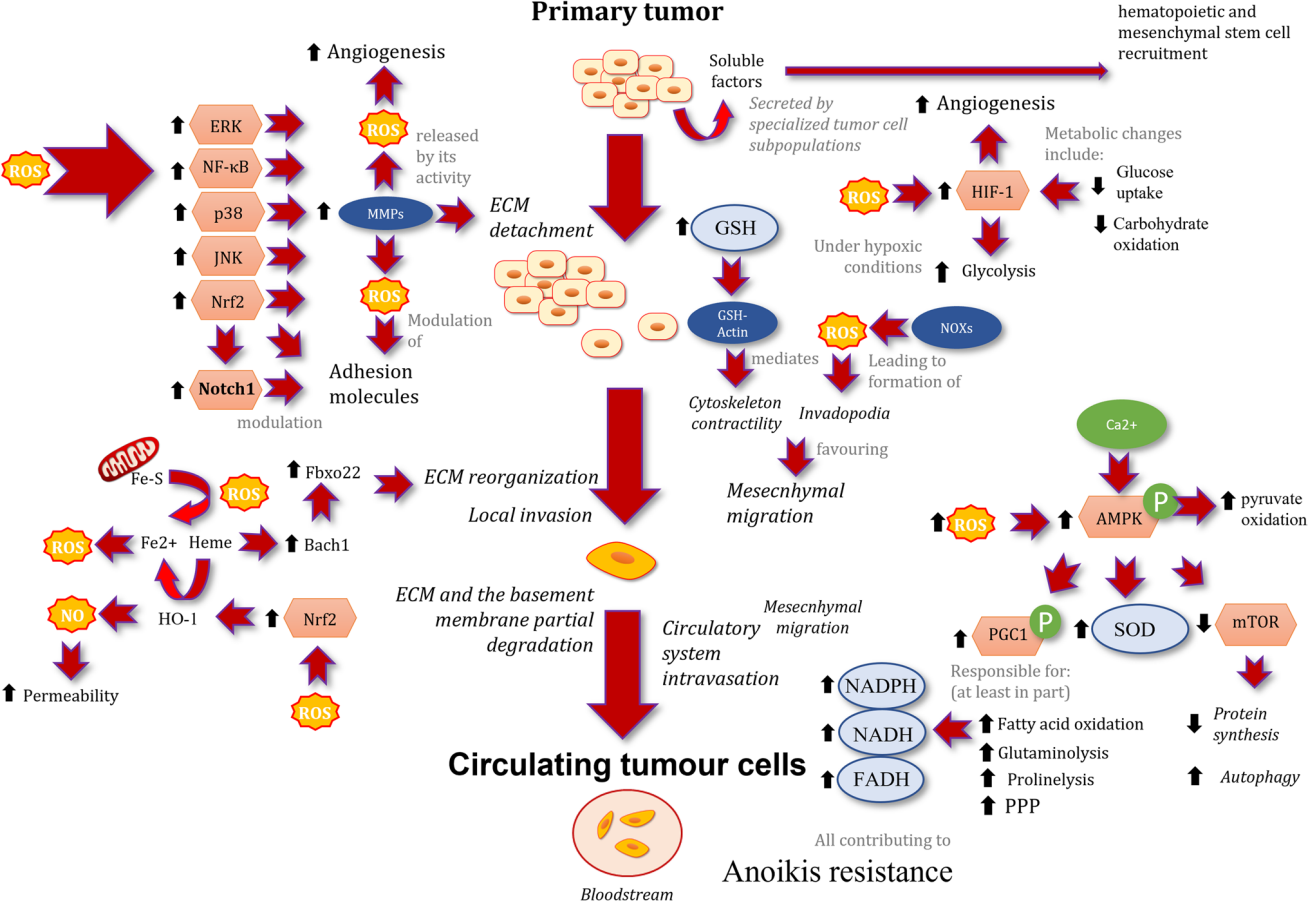
Intracellular ROS sources and redox signalling during epithelial to mesenchymal transition, extracellular matrix detachment, and extravasation of tumor cells. Black arrows indicate increases or decreases of activity or levels. Abbreviations: AMPK, AMP-activated kinase; Bach1, transcription regulator protein BACH1; ECM, extracellular matrix; ERK, extracellular-regulated kinase; Fbxo22, F-box only protein 22; Fe-S, ironsulphur proteins; GSH, reduced glutathione; HIF, hypoxia inducible factor; HO-1, heme oxygenase 1; JNK, c-Jun-aminoterminal kinase; MMP, matrix metalloproteinase; mTOR, mammalian target of rapamycin; NF-κB, nuclear factor κB; NOXs, NAD(P)H oxidases; Notch1, neurogenic locus notch homolog protein 1; Nrf2, nuclear factor erythroid 2-related factor 2; p38, p38 mitogen-activated protein kinase; PGC1, peroxisome proliferator–activated receptor γ coactivator-1; PPP, pentose phosphate pathway; ROS, reactive oxygen species; SOD, superoxide dismutase

During the initial phase of the metastasis cascade, namely tumour cell invasion and dissemination, a fraction of cancer cells requires undergoing morphological changes that allow them to invade the surrounding environment [119] (Fig. 1). These changes are thought to occur via a biological process which implies a loss of epithelial traits towards a gain of mesenchymal traits, named EMT [22]. Importantly, it has been described that EMT in tumour cells leads to distinct intermediary states, which represent a biological continuum between the two absolute phenotypes, epithelial and mesenchymal. These intermediary states are interconvertible and/or reversible given the epigenetic nature of the molecular mechanism that modulates the phenotypic transitions [120]. This genetic flexibility may be behind the higher adaptability to different microenvironments observed in DTCs. ROS are involved in several biological processes in cancer cells as cellular secondary messengers, playing an important role in EMT. Through different redox alterations, ROS can modify the biological function of proteins, which are sensitive to redox status of the cell. In addition to induce molecular and functional changes in proteins that are involved in the remodelling of the ECM, other proteins implicated in cytoskeleton remodelling, the establishment of cellular junctions and cell motility, may undergo variations which have a profound impact on metastatic invasion and dissemination [60, 121]. For instance, it has been observed that the knockdown of NADP-dependent malic enzyme (ME1), a major source of reducing equivalents, significantly decreased NADPH production and generated ROS, which decreased migration and invasion as well as altered EMT biomarkers expression. These data suggest that the promotion of EMT mediated by ME1 is driven in an ROS-dependent manner [121]. Furthermore, ME1 knockdown in human colon and lung cancer cells repressed cell growth under conditions of glucose starvation and induced senescence. In other words, ME1 knockdown diminished NADPH and induced high levels of ROS and apoptosis when subjected to stress conditions, for example, glucose starvation and anoikis [59].

### ECM detachment

Anoikis is a biological process defined as a caspase-dependent cell death mechanism which is induced by loss of integrin-mediated attachment to the ECM [122]. ROS modulation during anoikis has been studied through different experimental approaches. During matrix detachment, it has been observed a deficiency in glucose uptake that reduce pentose phosphate pathway (PPP) flux, diminishing NADPH and subsequently increasing ROS intracellular levels. However, the expression of the oncogene receptor tyrosine-protein kinase erbB-2 (ERBB2) has been shown to restore defective glucose uptake, rescuing PPP flux through the increase of glucose 6-phosphate. These changes lead to the generation of NADPH which fortified the antioxidant capacity of the cell [123]. In addition, cellular detachment from the ECM activated AMP-activated protein kinase (AMPK), which in turn limited NADPH consumption in fatty acid synthesis (FAS) and increased NADPH levels, through the production of fatty acid oxidation-induced NADPH. Therefore, tumour cells are capable to buffer the increase of intracellular ROS through the modulation of lipid metabolism and the maintenance of the intracellular levels of reducing equivalents, such as NADPH [124]. Subsequent studies have corroborated the direct effect of ECM detachment on AMPK activation. Interestingly, [125] found that AMPK was rapidly phosphorylated and upregulated in several cancer cell lines following matrix deprivation. Further investigations revealed that this early AMPK activation upon ECM detachment was triggered by an intracellular increase of ROS and Ca^2+^ levels and operated through the AMPK upstream activators serine/threonine-protein kinase STK11 (STK11) and calcium/calmodulin-dependent protein kinase kinase 2 (CaMKK2). Therefore, a Ca^2+^-ROS intracellular increase triggers the activation of the STK11/CaMKK-AMPK signalling cascade which promotes cell survival following ECM detachment. On the other hand, the activity of other antioxidant enzymes, such as catalase copper zinc superoxide dismutase (CuZnSOD) and MnSOD, in mitigating intracellular ROS levels to increase ECM-detached cell survival have been explored. Catalase and MnSOD, when antagonized, compromise the survival of detached mammary epithelial cells in a specific manner [126]. These observations were supported by another study indicating that overexpression and activity of MnSOD facilitate the survival of ECM-detached breast cancer cells and metastasis. Furthermore, the same study found a positive correlation between histologic tumour grade and expression of MnSOD in human breast cancer samples [106].

Additionally, the role of mitochondria and amino acid metabolism in tumour cell invasion and dissemination has been addressed through different experimental approaches. Lung carcinoma cells detached from the ECM-used isocitrate dehydrogenase (NADP) cytoplasmic (IDH1) to decarboxylate glutamine to citrate, which enters the mitochondria and participates in oxidative metabolism to provide energy to the cells. Concomitantly, but in the mitochondrial compartment, isocitrate dehydrogenase (NADP), mitochondrial (IDH2), synthesized NADPH, neutralizing mitochondrial ROS and stimulating cell survival [127]. Proline metabolism has also been shown to impact metastasis development. Proline has been found to support mitochondrial ATP production via proline dehydrogenase 1, mitochondrial (Prodh), which produces FADH2, a redox cofactor, and provides electrons to the electron transport chain, balancing redox homeostasis and enhancing metastasis formation [128].

### Ferroptosis

As mentioned before, there are other redox cellular changes that can lead to cell death due to ECM-detachment. A novel type of programmed cell death, known as ferroptosis, has been related to cell death induction in ECM-detached cells [129]. Ferroptosis is characterized by the accumulation of lipid hydroperoxides to lethal levels and in an iron-dependent manner [130]. From a biochemical perspective, lipid peroxides and other intracellular ROS such as superoxide (O2^−^), H_2_O_2_, and peroxyl radicals (ROO^•^) are generated through a cascade of reactions which involve different forms of ROS as intermediaries. O2^−^ is generated extracellularly or intracellularly by NADPH oxidase or the mitochondrial electron transport chain, respectively. In the mitochondria, O2^−^ produces ferrous iron (Fe^2+^) through its release from iron-sulfur (FE-S) or the reduction of ferric iron (Fe^3+^). O2^−^ can also be dismutated to H_2_O_2_ by superoxide dismutases in the mitochondria. H_2_O_2_ penetrate through membranes reacting with proteins and DNA, ultimately being detoxified by cellular peroxidases. ROO^•^, which originated from both the decomposition of ONOO^−^ and/or the reaction of H_2_O_2_ with Fe^2+^, initiates the lipid peroxidation cascade. ROO^•^ forms lipid radicals (L^•^) by reacting with lipids, which subsequently generate lipid peroxyl radicals through its reaction with oxygen. In a propagation reaction, lipid peroxyl radicals form lipid peroxides by reacting with polyunsaturated fatty acids (PUFAs) [131].

Sensitivity of the cells to ferroptosis has been associated with several biological mechanisms, such as the metabolism of amino acids, iron, and PUFAs, as well as glutathione, phospholipids, NADPH, and coenzyme Q10 biosynthesis. It has been shown that the induction of ferroptosis is driven by erastin, which is a small molecule that selectively inhibits the cystine/glutamate transport receptor (Xc^−^ system). Cell treatment with erastin inhibits the import of cystine and leads to the depletion of glutathione, finally causing the deactivation of the phospholipid hydroperoxide glutathione peroxidase (PHGPx). PHGPx acts converting lipid peroxides that might be toxic, into non-toxic lipid alcohols. Therefore, suppression of lipid peroxidation have been described to prevent ferroptosis in mammalian cells [130]. Furthermore, lipid peroxidation induced by ROS have been considered to play an important role in different types of cell death, based on chemical and structural alterations of the cell membranes, secondary to lipid peroxidation chain reactions [132]. In this line, the toxic products generated from lipid peroxidation have been shown to trigger apoptosis through distinct pathways, such as NF-kB [133], MAPK [134], and protein kinase C (PKC) [135], as well as autophagy via AMPK/mammalian target of rapamycin complex (mTORC) [136] and c-Jun-amino-terminal kinase (JNK)-apoptosis regulator Bcl-2 (Bcl-2)/Beclin-1 (BECN1) [134, 137]. Another study revealed the important protective role of Nrf-2 against lipid peroxidation and, consequently, ferroptosis. Nrf-2 is a key regulator of the cellular antioxidant response and controls the expression of genes that code proteins which catalyze reactions leading to oxidative and electrophilic stress neutralization [138]. In particular, the expression of the Xc^−^ system and PHGPx, which play a prominent role in ferroptosis inhibition, is under the control of Nrf-2.

Taken together, these studies exemplify a well-known fact in tumour biology, which is that the net effect of intracellular ROS can either promote tumour cell endurance through the activation of pro-survival signalling pathways or induce cell death, if the intracellular level increases beyond a lethal level that cannot be quenched by the several antioxidant mechanisms. Therefore, in order to survive, tumour cells might reach a situation of equilibrium through which they favor the intracellular raise of ROS, at sub-lethal levels, to activate signalling pathways that promote several biological processes involved in tumour progression. At the same time, the intracellular raise of ROS might be maintained at non-toxic levels through the action of the antioxidant system. This non-physiological redox balance might confer survival advantages to tumour cells and be at the base of the metastatic behavior [139, 140].

## Infiltration of CTCs and DTCs fate

**Fig. 2:**
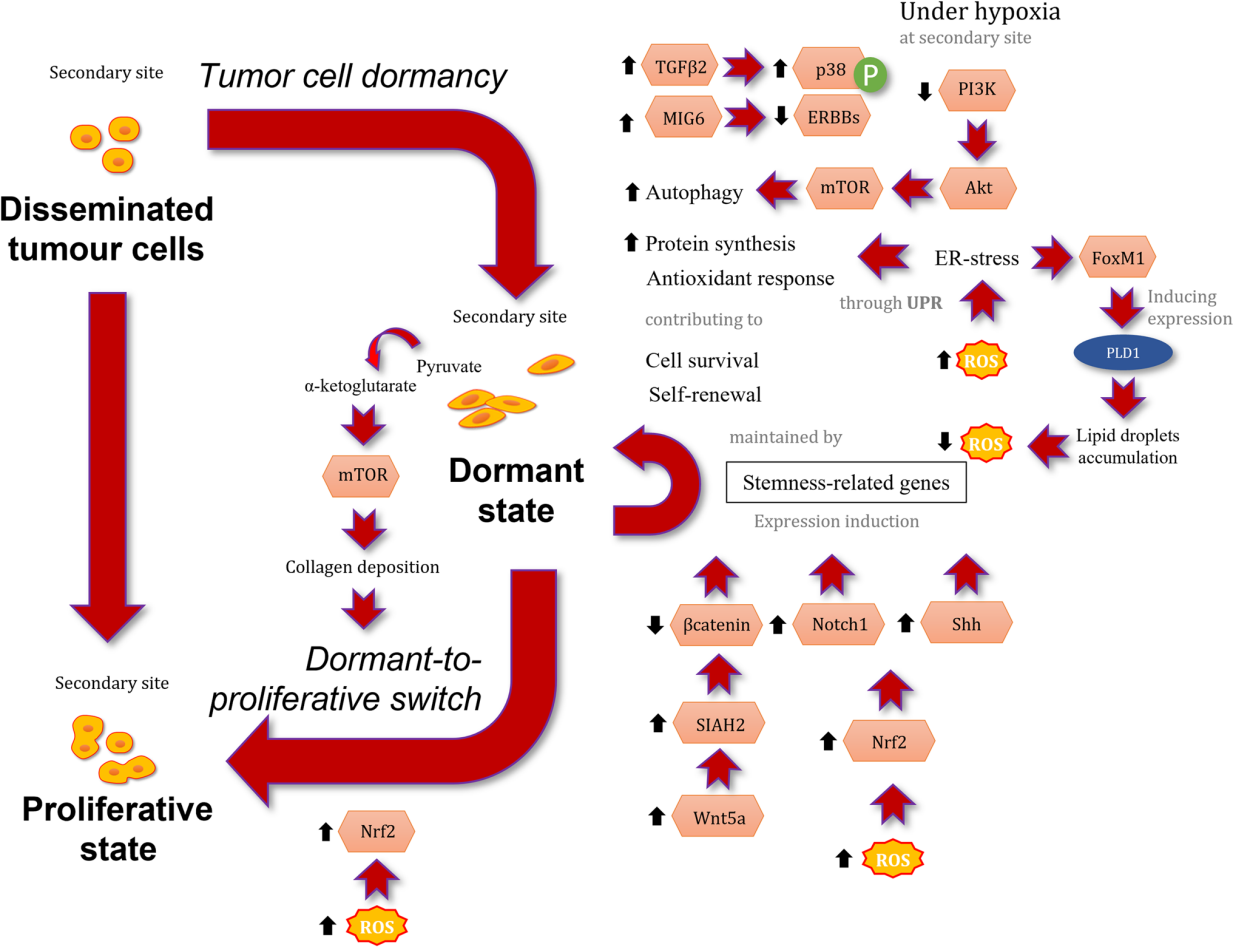
Intracellular ROS sources and redox signalling in disseminated dormant tumour cells. Black arrows indicate increases or decreases of activity or levels. Abbreviations: Akt: protein kinase B; ECM, extracellular matrix; ERBBs, epidermal growth factor (EGF) and its receptors; FoxM1, forkhead box M1; HIF, hypoxia inducible factor; PLD1, phospholipase D1; MIG6, mitogen-inducible gene 6 protein; mTOR, mammalian target of rapamycin; Notch1, neurogenic locus notch homolog protein 1; Nrf2, nuclear factor erythroid 2-related factor 2; p38: p38 mitogen-activated protein kinase; PI3K, phosphoinositide 3-kinase; ROS, reactive oxygen species; SIAH2, SIAH2 E3 ubiquitin-protein ligase; Shh: sonic hedgehog; TGFβ2, transforming growth factor-beta 2; Wnt5a, protein Wnt-5a

The adhesion of CTCs to endothelial cells is considered the first step of the extravasation. Following migration through endothelial parenchyma, cancer cells may reach a distant organ, becoming DTCs. At this point, a substantial proportion of DTCs undergo some form of cell death due to the several biological hurdles encountered at this phase of the metastatic cascade. A minor fraction of the metastatic cells which disseminated from the primary tumour will either enter a dormant or a proliferative state, in accordance with several intracellular and extracellular signals [141, 142] (Fig. 2).

### ECM reattachment

In determining cell fate when entering the secondary sites, the re-adhesion of tumour cells to the ECM at the metastatic niche is known to be rate-limiting step [143]. The biophysical properties of the ECM have been related to tumour cell antioxidant homeostasis. A recent study [144] has shown that breast cancer cells cultured on a soft ECM increase the production of mitochondrial ROS (mtROS) due to changes in mitochondrial dynamics, driven by an increase in peri-mitochondrial F-actin, which was found to control dynamin-1-like protein (DRP1) activity and mitochondrial fission. These changes conferred enhanced tolerance to oxidative stress through Nrf-2 antioxidant transcriptional response to finally provide breast tumour cells with a survival advantage. Interestingly, the activation of the F-actin/DRP1/Nrf-2 axis was shown to be upregulated in breast tumour dormancy models where inhibition of DRP1 and Nrf-2 prevented the dormant-to-proliferative switch, showing the deep impact of mitochondrial dynamics and redox signalling in DTC fate and metastasis.

Not only the biophysical properties of potential secondary sites have a profound impact on DTCs fate, but biochemical and metabolism-derived products released to the tissue stroma determine the adoption of different survival strategies from DTCs. In this line, an enrichment of pyruvate in the lung stroma has been shown to enforce the adaptation of breast DTCs through the conversion of pyruvate to α-ketoglutarate catalyzed by mitochondrial alanine aminotransferase 2 (ALT2). The raise in the extracellular concentration of α-ketoglutarate potentiated mTORC1 signalling and supporting the remodelling and deposition of collagen [145], a known inducer of the dormant-to-proliferative switch and metastasis formation [146]. Indeed, metabolic rewiring has been shown to discern between micro- and macrometastasis. Using patient-derived-xenograft (PDX) models of breast cancer, Davis et al. [147] showed that the expression of genes involved in mitochondrial oxidative phosphorylation (OXPHOS) was upregulated in micrometastasis as compared to primary tumour cells, where expression of glycolytic enzymes were highest. The authors corroborated these findings through metabolomic data and pharmacologic inhibition of OXPHOS, which further demonstrated its functional importance in the context of metastasis since OXPHOS blockade significantly reduced metastatic burden in the lungs of their PDX model. Finally, Viale et al. [148] showed that oncogene ablation, using an inducible model of KRAS-mutant pancreatic ductal adenocarcinoma (PDAC), selected a subpopulation of dormant PDAC cells which displayed stem cell features and were highly dependent on OXPHOS for survival. In addition, transcriptomic and metabolic analyses performed in surviving cells revealed major expression of genes regulating autophagy and mitochondrial and lysosome activity. Considering that mitochondrial OXPHOS is a major source of intracellular ROS [149] and that OXPHOS may be a commonly activated pathway in quiescent or dormant cancer cells, the raise in intracellular ROS and its consequences in dormant DTC signalling and survival warrants further investigation.

### Hypoxia

Hypoxia, defined as a biophysical condition of the cellular microenvironment by which a tissue is not able to provide sufficient oxygen to sustain homeostasis [150], has been linked to DTC fate, as it is known to significantly impact tumour progression, resistance to the treatment, and metastasis. Indeed, hypoxia is a poor-prognosis feature of solid tumours [151]. Hypoxia signalling has been shown to regulate diverse steps of the metastatic cascade: EMT, invasion, intravasation, survival of CTCs, extravasation, establishment of the premetastatic niche, and proliferation from micrometastasis to macrometastasis [152]. Under low oxygen, HIF is stabilized though the inhibition of its degradation by the von Hippel-Lindau disease tumour suppressor (pVHL), consequently being translocated to the nucleus where it promotes the expression of genes that lead to the adaptation to the low levels of oxygen [153]. Fluegen et al. [154] showed that a hypoxic microenvironment induce the reversible upregulation of hypoxia (glucose transporter 1 (GLUT-1), HIF1α) and dormancy (COUP transcription factor 1 (COUP-TF1), differentiated embryonic chondrocyte gene (DEC2), p27) genes in primary breast and HNSCC. Remarkably, post-hypoxic DTCs were characterized by a dormant phenotype and high expression of COUP-TF1, DEC2, p27, and TGFβ2. Upon hypoxia, COUP-TF1 and HIF1α were shown to induce the expression of p27 and quiescence in tumour cells. However, dormant DTCs did not show a high-level expression of other hypoxia markers, such as GLUT-1. These data suggested that the preservation of the dormant phenotype in post-hypoxic DTCs was independent of GLUT-1 expression or that additional hypoxia-responsive pathways may contribute to the maintenance of dormancy in DTCs. In addition, following hypoxia exposure, T-Hep3 cells in the lung significantly overexpressed TGFβ2 as compared to proliferating metastases, where its signalling was silenced. In this line, the authors suggested that post-hypoxic DTCs home to TGFβ2 expressing metastatic niches that may contribute to dormancy induction. Indeed, Bragado et al. [155] showed that TGFβ2 signalling in the bone marrow activates MAPK p38α/β, lowering the ERK/p38 ratio, and thus, inducing dormancy in T-Hep3 DTCs *in vivo*. The same signalling axis has been shown to induce tumour dormancy in a model of prostate cancer [156].

In line with these findings, Erler et al. [157, 158] found that Lysine Oxidase (LOX), a protein of the ECM, is controlled by hypoxia and HIF. Under hypoxic conditions, breast cancer cells in the primary tumour secrete LOX, which accumulates in the premetastatic niche and modifies the secondary site ECM. LOX crosslinks collagen IV at the basement membrane, which is essential for the recruitment of CD11b + myeloid cells and secretion of MMP2. In turn, CD11b + myeloid cell-derived MMP2 cleaves collagen at the pre-metastatic niche, which promotes the invasion of bone marrow-derived cells (BMDCs) and metastatic cells. Indeed, LOX inhibition significantly decreased metastatic growth. In addition, hypoxia has also been shown to induce a quiescent state in a model of patient-derived primary lung cancer harboring activating EGFR mutation [159]. Mechanistically, hypoxia upregulated the expression of mitogen-inducible gene 6 protein (MIG-6) a negative regulator of ERBB signalling, which prevented heterodimer formation of ERBB family receptor tyrosine kinases (RTKs) and downstream signalling inducing tumour cell quiescence and resistance to EGFR tyrosine kinase inhibitor (TKI) treatment. Interestingly, when MIG-6 was downregulated in mutant EGFR lung cancer cells under hypoxic conditions, EGFR signalling was restored, promoting the phosphorylation of ERK and AKT through increased EGFR-HER3 binding, as well as their sensitivity to EGFR-TKI and radiation. In accordance with this results, analyses of tumour samples from lung cancer patients with EGFR mutations revealed a significant inverse correlation between MIG-6 expression their survival rates after the treatment with EGFR-TKI [159].

## Redox signalling in tumour cell dormancy and the dormant-to-proliferative switch

After dissemination, cancer cells might enter dormancy. As it has already been shown, redox signalling plays a crucial role in earlier steps of the metastatic cascade, and it continues having a deep impact in the onset and maintenance of a dormant phenotype and the survival of quiescent DTCs. Current bibliography suggests that dormant DTCs activate a series of molecular mechanisms aiming to control excessive oxidative stress, given that they appear to be highly sensitive to intracellular ROS while in a dormant state. Oxidative stress has been found to modulate tumour cell dormancy through the inactivation of Kelch-like ECH-associated protein 1 (Keap1) and translocation of Nrf-2 into the nucleus, leading to the promotion of neurogenic locus notch homolog protein 1 (Notch1) and sonic hedgehog protein (SHH) transcription and subsequently activating downstream signalling [160, 161]. Together with Notch1 and SHH-initiated signalling pathways, Wnt signalling may sustain DTC dormancy through transcription of stemness-related genes [162]. The important role of Wnt signalling in dormancy maintenance has been suggested by additional studies. Protein Wnt-5a (Wnt5a) expression in the osteoblastic niche has been found to promote dormancy in a model of prostate cancer via the activation of the Wnt5a/tyrosine-protein kinase transmembrane receptor ROR2 (ROR2)/E3 ubiquitin-protein ligase SIAH2 (SIAH2) signalling axis [163]. This study showed that the reversible induction of dormancy was driven by Wnt5a through enhanced expression of SIAH2, which in turn repressed Wnt/β-catenin signalling. Besides, this mechanism of dormancy induction was showed to depend on ROR2, whose expression was inversely correlated with the disease-free survival (DFS) rates in prostate cancer patients who developed bone metastasis. Finally, the disrupting effect of itraconazole, a Wnt inhibitor, was evaluated in a patient-derived colorectal cancer model. The authors observed an increased cytotoxic vulnerability in dormant colorectal cancer cells exposed to itraconazole, supporting the role of Wnt in the maintenance of a dormant phenotype in DTCs [162].

As it has been mentioned before, cell stress has a profound impact in the modulation of molecular signalling pathways leading to tumour cell dormancy. Two stress-related processes have been recently studied regarding dormant DTC survival and maintenance: autophagy and unfolded protein response (UPR).

### Autophagy

Autophagy is an evolutionarily conserved physiological mechanism involved in the maintenance of cell homeostasis under metabolic stress [164]. This physiological mechanism can be hijacked by tumour cells to survive the numerous stresses encountered along the metastatic cascade. In this regard, Vera-Ramirez et al. [58] reported that breast DTCs activate autophagy via BECN1-independent non-canonical pathway to survive upon the establishment of a cell dormancy program both *in vitro* and *in vivo*. Pharmacological inhibition (through administration of different autophagy inhibitors, such as bafilomycin, 3-methyladenine, and hydroxychloroquine) or genetic (through knockdown of autophagy related 7 (*ATG7*) but not *BECN1*) means dramatically impaired the survival of dormant breast cancer cells. Mechanistically, autophagy inhibition resulted in the intracellular accumulation of depolarized mitochondria and toxic oxidative by-products which drove apoptotic cell death. Interestingly, quenching of mitochondria-derived ROS rescued cell viability, further showing the crucial importance of ROS management in the survival of dormant DTCs. Additionally, autophagy has been shown to be activated under hypoxic conditions and decrease intestinal inflammation through the downregulation of the mTOR/NOD-like receptor, pyrin domain-containing 3 (NLRP3) pathway [165]. Moreover, whereas the inhibition of the PI3K/AKT/mTOR and MAPK signalling pathways resulted on autophagic cell death [166], lower levels of PI3K in association with microenvironmental factors were correlated with the promotion of autophagy-induced dormancy in ovarian cancer [167]. These data highlight the hormetic and environment-dependent net effect of the activation of the molecular networks related to redox signalling and autophagy. On the other hand, oxidation of ATG4, ATG7, and ATG3 have also been found to decrease autophagy. A study revealed that ATG4B activity was reversibly modulated by the formation of intramolecular disulfide bonds in response to oxidative stress. Modifications were described in Cys292 and Cys361 residues of the ATG4B protein [168]. Furthermore, inactive ATG3 and ATG7 are covalently bond to microtubule-associated protein 1A/1B-light chain 3 (LC3) and protected from oxidation. When activated, LC3 is transferred to phosphatidylehanolamine, the covalent interaction is lost, and the catalytic thiols of both ATG3 and ATG7 are exposed to inhibitory oxidation, preventing LC3 transfer and autophagy [169].

### Unfolded protein response

The accumulation of unfolded or misfolded proteins in the ER lumen is a salient feature of specialized secretory cells leading to a cellular condition known as endoplasmic reticulum (ER) stress. ER stress is counteracted by the activation of the unfolded protein response (UPR), which is a homeostatic signalling network that either promote the recovery of ER function or potentiates apoptosis, depending on the duration and intensity of the stress stimuli. Chronic UPR has been shown to be involved in the pathogenesis of a wide variety of human diseases, from diabetes to cancer, highlighting the role of this highly interconnected signalling network as a stress “dimmer” in the cell, beyond ER stress and protein folding [170–172]. Indeed, some types of cellular stress and extracellular stress signals, such as hypoxia, activate p38 MAPK which can in turn inhibit the expression of Forkhead Box protein M1 (FoxM1), Proto-Oncogene C-Jun (c-Jun), and the uPAR transcripts, supressing the activation of ERK and triggering downstream UPR signalling [173]. An analysis revealed the upregulation of ER-stress response genes in HEp3 cells under p38 regulation: binding immunoglobulin protein (BiP, also known as GRP-78), protein disulfide-isomerase (PDI)/protein disulfide-isomerase A3 (PDIA3, also known as ER60), serpin H1 (HSP47), and cyclophilin B [174]. The data showed that under normal conditions, BiP is bound to domains located in the ER-luminal kinase receptors protein kinase R-like endoplasmic reticulum kinase (PERK), serine/threonine-protein kinase/endoribonuclease IRE1 (IRE1α), and activating transcription factor 6 (ATF6), among others. However, in the presence of misfolded proteins, BiP dissociated from the protein complexes and translocated to the ER lumen activating PERK, IRE1α, and ATF6. When active, PERK phosphorylated eukaryotic translation initiation factor 2A (eIF-2A) which inhibited eIF-2A-dependent protein synthesis and induced ATF4 to upregulate the expression of genes associated with protein synthesis and antioxidant response. The activation of this molecular cascade promoted cell survival and growth arrest. PERK has also been considered to induce the activation of the Nrf-2 transcription factor, to inactivate C/EBP-homologous protein (CHOP) and block apoptosis [175]. Activated IRE1α promoted the spliced form of X-box-binding protein 1 (XBP-1) (XBP-1 s), which is a transcription factor that binds to chaperones and endoplasmic-reticulum-associated protein degradation (ERAD) gene promoters to modify or degrade misfolded proteins. IRE1α can stimulate Grp78/BiP to block apoptosis regulator BAX (Bax)-elicited proapoptotic signals. Last, ATF6 has also been shown to promote cell survival in HEp3 cells through the activation of mTOR signalling, controlled by GTP-binding protein Rheb (Rheb) [176–178].

Therefore, the UPR-mediated transcriptional induction of genes that increase protein folding capacity and antioxidant machinery is an important mechanism to re-establish cellular homeostasis and to alleviate protein folding stress [176, 177]. In addition, protein post-translational modifications, which are key for appropriate folding, are frequently highly sensitive to the redox status of the cell. The formation of disulfide bridges is catalyzed by protein disulfide isomerases and other oxidoreductases which, in turn, contribute to ROS generation and redox unbalance in the ER [172, 178]. Evidence suggests that ROS production and oxidative stress are not only coincidental to ER stress. Rather, ROS are an integral part of the UPR signalling network, supporting either proapoptotic or proadaptive UPR signalling [170–172].

### Intracellular molecular mechanisms

Regarding to the onset of proliferation following quiescence, several lines of evidence support the role of the microenvironment in triggering the dormant-to-proliferative switch of DTCs in the metastatic niche, as reviewed in previous sections of this manuscript. On the contrary, our knowledge about the intracellular molecular mechanisms contributing to the dormant-to-proliferative switch is much more limited, although redox signalling is thought to play an important role in the reactivation of dormant DTCs. For instance, Nrf-2 has been shown to be upregulated upon downregulation of ERBB2 in breast cancer cells and secondary to an increase in intracellular ROS due to altered cellular metabolism. It has also been reported that Nrf-2 was activated during dormancy and its signalling has been found to induce the re-establishment of redox homeostasis in recurrent tumours and upregulation of *de novo* nucleotide metabolism, ultimately promoting the reactivation of dormant breast cancer cells [179].

It has also been suggested that lipid metabolism is relevant to this step of the metastatic cascade. Pascual et al. [180] showed that a sub-population of metastasis-initiating oral carcinoma cells (defined by overexpression of CD44) expressed high levels of the fatty acid receptor CD36 and lipid metabolism genes. Supplementation with palmitic acids or a high-fat diet fuelled tumour proliferation and metastatic outgrowth *in vitro* and *in vivo*, respectively. Blocking CD36 dramatically inhibited the formation of metastasis *in vivo*. These data showed that metastatic cells rely on lipid metabolism in early steps of the metastatic colonization and growth.

Remarkably, a series of experiments using organoid cultures and mouse models revealed that tumour recurrent cells showed increased ROS intracellular levels and modified lipid metabolism. When the synthesis or intramitochondrial transport of fatty acids was inhibited, the levels of cellular ROS and DNA injury decreased, promoting tumour cell proliferation. Moreover, a significant reduction on the rate of *in vivo* recurrence was achieved through NAC-dependent ROS scavenging and proliferation inhibition through treatment with progesterone antagonist [181]. Interestingly, Carracedo et al. [182] unveiled the role of promyelocytic leukemia (*PML*) gene as an inhibitor of peroxisome peroxisome proliferator-activated receptor gamma coactivator 1-alpha (PGC-1-α) acetylation and as a potent activator of peroxisome proliferator-activated receptors (PPAR) signalling and fatty acid oxidation in breast cancer. PML expression promoted tumour cell invasion *in vitro* and was correlated with poor prognosis in breast cancer patients. Therefore, fatty acid oxidation and fatty acid metabolism have been shown recurrently to critically modulate metastasis development. Lipid metabolism is intimately ligated to redox signalling, both as a ROS source through their catabolism [183] and as a ROS intracellular scavenger when stored as cytoplasmatic lipid droplets (LDs) [184]. In the context of metastasis, the antioxidant role of intracellular lipids stored in the form of LDs has been shown to protect DTCs against paclitaxel-induced ROS production and promote breast cancer metastasis. Mechanistically, metastasis competent cells overexpressed FoxM1, whose transcriptional activity stimulated the expression of phospholipase D1 (PLD1), which in turn, promoted LD accumulation [168]. These innovative approaches and data underscoring the antioxidant and protective role of lipids, beyond their traditional metabolic and signalling roles, open new research avenues in our understanding of dormant DTC biology and metastatic relapse.

## Redox signalling during colonization and metastatic outgrowth

**Fig. 3:**
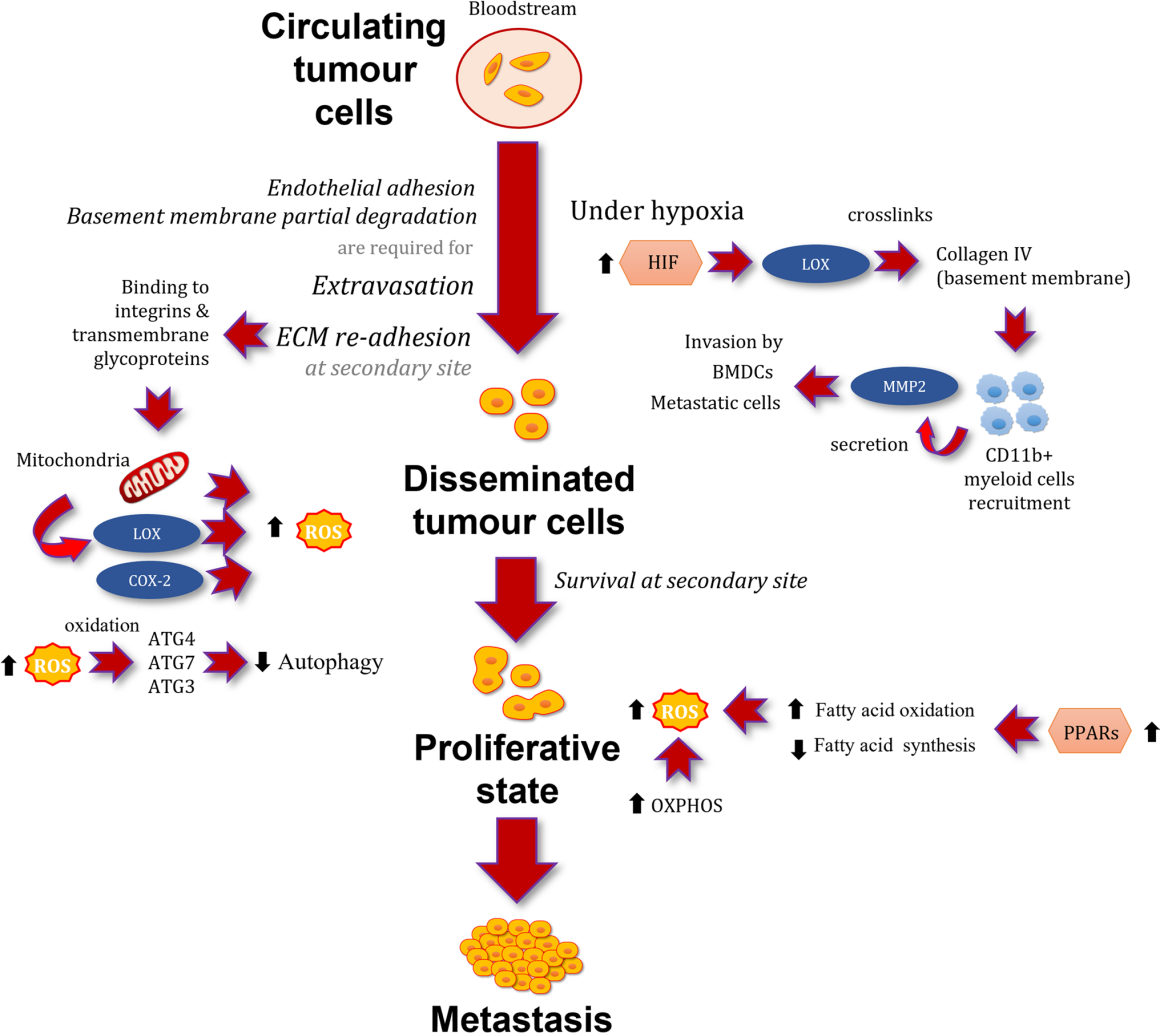
Intracellular ROS sources and redox signalling in the dormant-toproliferative switch and the metastatic outbreak of disseminated tumor cells. Black arrows indicate increases or decreases of activity or levels. Abbreviations: ATG3, autophagy related 3; ATG4, autophagy related 4; ATG7, autophagy-related 7; BMDCs, bone marrow-derived cells; COX-2, ciclooxygenase-2; ECM, extracellular matrix; HIF, hypoxia-inducible factor; LOX, lysyl oxidase; MMP2, matrix metalloproteinase 2; OXPHOS, oxidative phosphorylation proteins; PPARs, peroxisome proliferator–activated; ROS, reactive oxygen species

The functional contribution of ROS to metastasis formation and metastatic outgrowth has been studied deepest as compared to any other phase of the metastatic cascade [111] (Fig. 3). During some stages, cancer cells have higher basal ROS levels than normal cells, but these increases can be transient since cancer cells can increase its antioxidative capacity to adapt itself to excessive ROS conditions. Moreover, cancer cells are known to induce mechanisms that limit oxidative damage and repair cellular structures damaged by ROS, all of which endorses them with a higher oxidative stress tolerance. However, certain ROS level over the cytotoxic threshold could selectively kill cancer cells by different mechanisms. As mentioned, ROS plays an essential role in the cellular signalling that regulates cell proliferation and cell survival, but chronic oxidative stress situations with maintained higher levels of ROS could result in the disruption of homeostasis and cell death. However, it is very difficult to establish a ROS threshold defining cell fate, in part as consequence of difficulties measuring ROS levels at real time and cell redox state. In addition, exposure of *in vitro* and *in vivo* models to oxidative stress is difficult to time and modulate since ROS exposure results in oxidative chain reactions that expand intracellularly and cannot be controlled experimentally. Taking into account these technical considerations, the development of redox biosensors is warranted.

### The hypoxia and HIF signalling pathway

As mentioned before, the tissue microenvironment is known to play a pivotal role in cancer metastasis. A widespread characteristic of tumours is their inability to develop adequate blood vessels with the consequence being relative hypoxia. Hypoxic conditions stimulate blood vessel development, whereby the blood flow in these new vessels is often chaotic, causing oxidative stress [77]. Consequently, within a growing tumour mass, cancer cells repeatedly face cycles of hypoxia and reoxygenation [85]. H_2_O_2_ secreted by cancer cells has been proposed to mimic the effects of hypoxia under aerobic conditions in adjacent fibroblasts and other stromal cells, resulting in production of ROS, which has been shown to stimulate angiogenesis. For instance, it has been shown that the administration of Mn(III) ortho-tetrakis-N-ethylpyridylporphyrin, a potent scavenger of ROS and RNS, attenuates angiogenesis *in vivo*, modifies the density of microvessels, and decreases the proliferation rate of endothelial cells in a mouse model of breast cancer [185].

Moreover, glucose deficiency is one of the main factors leading to oxidative stress in tumours, either in the stage of tumour initiation or progression [186]. In relation to metastasis, it is important to note that detaching from ECM leads to ATP deficiency owing to a reduction of glucose transport [123], which would lead to a strong induction of ROS production. VEGF expression can be regulated by nutrient deprivation and hypoxia, which both increase intracellular levels of ROS [187]. Increased generation of H_2_O_2_ also led to accumulation of HIF-1α [188]. MnSOD suppresses the induction of HIF-1α in human breast carcinoma cells [188]. Likewise, suppression of endogenous ROS by mitochondrial inhibitors or GPx decreased HIF-1 induction and VEGF expression in cancer cells [189]. Moreover, it has been reported in three different tumour mouse models that antioxidants, as NAC and vitamin C, exert their antitumoural effect mainly acting on HIF-1α level [190]. These results suggest that both, superoxide and H_2_O_2_, may contribute to HIF-1α accumulation. At moderate levels, ROS induce transcription of *HIF1A* gene and stabilize the encoded protein by inhibiting the activity of the iron-dependent prolyl 4-hydroxylase (4-PH) as a consequence of catalytic Fe^2+^ oxidation of Prolyl hydroxylase domain-containing protein 2 (PHD2) [191]. This mechanism seemed involved in physiological stabilization of HIF under mild (1–3% O_2_), but not deep, hypoxia, a condition reportedly accompanied by mitochondrial production of ROS [192, 193]. This is probably mediated by an increase in basal levels of H_2_O_2_ and superoxide, due to decreased expression of several antioxidant enzymes such as peroxiredoxins (Prxs) and CuZnSOD [194]. Moreover, S-nitrosylation of HIF at Cys-800 has been shown to increase factor stability and transcriptional activity, likely by promoting HIF binding to p300 [195]. Interestingly, PH inhibition by ROS may contribute to normoxic accumulation of HIF in cancer cells [196, 197].

As a consequence of hypoxia-induced HIF-1α activation, HIF-1 and its cofactor p300 induced the expression of pro-angiogenic genes [198], such as VEGF and VEGF receptors [199, 200]. HIF-1 stabilization is important for cancer metastasis due to several reasons. Hypoxia, through HIF activation, drives cancer cell invasion and metastatic progression in various cancer types. In epithelial tumours, hypoxia induces the transition to amoeboid cancer cell dissemination. Accordingly, hypoxia triggers EMT in several human tumour cells through the generation of mitochondrial ROS which correlated with elevated intracellular levels of HIF [201]. Invasion and metastasis in cells where metastasis was promoted by the generation of mitochondrial ROS as consequence of mitochondrial DNA mutations have been also associated with elevated intracellular levels of HIF, suggesting a direct link between ROS and the pro-invasive program triggered by this transcription factor [93].

Moreover, HIF-1 regulates glycolysis-related genes and inhibits mitochondrial respiration resulting in metabolic adaption of tumour cells to hypoxia [202, 203]. Likewise, HIF-1 prevents intracellular acidification, which leads to an increased formation of lactate and CO_2_ [202], both compounds favoring ECM degradation and cell invasion [204]. In addition, HIF-1α-induced promotion of metastasis and EMT in osteosarcoma cells has been shown to depend on a reduction of intracellular ROS production. In osteosarcoma (OS) cells under hypoxic conditions, HIF-1α induces the overexpression of the mitochondrial NADH dehydrogenase (ubiquinone) 1 alpha subcomplex 4-like 2 (NDUFA4L2) protein, which in turn promoted OS cell migration, invasion, proliferation, and EMT. Drug-and genetically-induced inhibition of the NDUFA4L2 protein negatively impacted OS cell survival, which could be in part compensated through a reduction in intracellular ROS levels through autophagy activation [205].

Along with oxygen availability, interstitial fluid hydrostatic pressure that generally increases with the rapid growth of malignant tumours also has been shown to influence HIF activity. Higher protein levels of HIF‑1α have been found in Lewis lung cancer (LLC) cells that were exposed to 50 mmHg hydrostatic pressure for 24 h. This condition was associated with upregulation of numerous metastasis‑promoting genes (hepatocyte growth factor (*HGF*), cadherin 11 (*CDH11*), and ephrin type-B receptor 2 (*EPHB2*)) and the downregulation of metastasis suppressing genes (KiSS-1 metastasis suppressor (*KISS1*), spleen associated tyrosine kinase (*SYK*), and HIV-1 Tat interactive protein 2 (*HTATIP2*)). Thus, elevated interstitial fluid hydrostatic pressure in malignant tumours may promote the onset of the metastatic cascade by stabilizing HIF‑1α [206]. Moreover, it was found that the exposure of the LLC cells to high hydrostatic pressure, prior to intravenous injection into healthy mice, resulted in higher cell survival/retention in the lungs 24 h later and resulted in more metastatic tumour lesions 4 weeks later [206].

In the previous model, elevated HIF‑1α levels also correlated with the levels of the antioxidant enzymes, CuZnSOD and MnSOD, improving tolerance to oxidative stress in LLC cells exposed to high hydrostatic pressure. These data suggested that metastasis promotion in cancer cells exposed to high hydrostatic pressure would be driven by oxidative damage prevention through HIF transcriptional activity [206]. In addition, under hypoxic conditions, HIF-dependent upregulation of the one-carbon metabolism enzyme serine hydroxymethyltransferase, mitochondrial (SHMT2), promotes mitochondrial serine catabolism and NADPH production [207], to raise the antioxidant capacity and prevent ROS-mediated tumour cell death [207].

### Small GTPases

The Rac1 GTPase has important functions in ROS-mediated actin reorganization of migrating tumour cells [208, 209]. Intracellular ROS increases are involved in MMP-3–induced EMT in murine breast epithelial cancer cell lines which, concomitantly, correlated with increased expression of a Rac1b splicing variant. It has been suggested that the treatment with MMP-3 stimulates the expression of Rac1b that would be responsible for increasing ROS levels [73]. Importantly, a cell shape change is required for EMT induction by MMP3 and Rac1b [210], in keeping with the notion that Rac-dependent ROS transduce mechanical perturbations into a pro-invasive gene expression program [76]. Secretion and activation of MMP-2 has been reported to be dependent on Rac1 and ROS generation in pancreatic carcinoma PANC-1 cells exposed to EGF. Moreover, it has been suggested that signalling events downstream of EGF receptor involved PI3K- and Src-dependent activation of Rac1 [211]. ROS are in turn responsible for the activation of the SNAI1 factor, a key transcriptional inducer of the EMT program [212–214]. In addition, synergistic signalling between growth factors and integrins leads to an intracellular oxidative burst through a Rac1-dependent increase in mitochondrial ROS production [215, 216]. In this sense, Rac1-mediated ROS generation was involved in prometastatic signalling through c-Met, one of its upstream regulators, in a murine model of invasive melanoma [217]. The redox cascade triggered by overexpression of c-Met involved generation of H_2_O_2_ via CuZnSOD-mediated dismutation of superoxide. Importantly, tumour cell capacity to form experimental lung metastases *in vivo* was inhibited after blocking the abovementioned cascade [217].

Some studies also have demonstrated the involvement of Rac signalling in cytoskeletal rearrangement and in mediating integrin signalling. ROS generated by Rac1-induced NOX have been shown to activate the cofilin pathway and, thus, to contribute to increased cell migration [90, 91]. Conversely, a density-dependent decrease of NOX-Rac1 activity and ROS production in response to receptor tyrosine kinases engagement mediates growth inhibition by cell–cell contact in fibroblasts [218]. Interestingly, it has been reported that mesenchymal and amoeboid motility styles are interconvertible through the reciprocal regulation of Rac and Rho GTPases in melanoma cells [219]. Moreover, activation of Rac and subsequent generation of ROS leads to NF-κB activation and MMP-1 production in response to integrin-mediated cell shape changes [216]. Rac1-mediated changes in cellular ROS levels also increase the migratory potential of MCF-7 and T47D breast cancer cells probably through NF-κB [220]. Lastly, increased activity of Rac1 in primary endothelial cells has been shown to mediate the loss of cell–cell adhesions and loosens the integrity of the endothelium, which allows the intravasation of cancer cells [221].

### Protein kinases

#### The PI3K/AKT signalling pathway

In general, phosphatases as protein-tyrosine phosphatase 1B (PTP-1B), SH2 domain-containing PTP (SHP-2), phosphatase and tensin homolog (PTEN), and low molecular weight phosphotyrosine protein phosphatase (LMW-PTP) are inhibited by ROS [222], whereas kinases may be inhibited or activated [223]. In particular, ROS activate nonreceptor protein kinases belonging to the Src family; small G proteins, such as Ras; and other protein tyrosine kinases including tyrosine kinase receptors of growth factors [224–226]. Some studies have demonstrated that H_2_O_2_ can promote the activation of Ras and growth factor signalling which in turn activates PI3K/AKT pathway and inactivates PTEN signalling cascades. Phosphatases are transiently inactivated by H_2_O_2_ in the context of growth factor, cytokine or integrin signalling, as a necessary step for the propagation of tyrosine phosphorylation cascade. Targets for mitochondrial ROS in these processes are SHP-2 and FADK 1, while cytosolic ROS target the phosphatases LMW-PTP and SHP-2, receptor tyrosine kinases, Src-family kinases, FADK 1, and structural proteins such as β-actin [84]. Activation of phosphatases and Src occurs through direct oxidation, whereas activation of FADK 1 is probably indirect through upstream signalling events leading to its tyrosine phosphorylation [227]. The tyrosine kinase Src (that leads to activation of MAPK, ERK, and AKT) also regulates NOX1-induced generation of ROS [87]. Both Src and FADK 1 are initiators of focal adhesion formation in adherent cells, contributing to cell spreading, cell migration, and prevention of anoikis.

Specifically, through the formation of disulfide bonds between catalytic cysteines, H_2_O_2_ inactivates PTEN and unlocks the PI3K-dependent recruitment of its downstream kinases, such as AKT [228] contributing to increasing cell adhesion to ECM, cell spreading, and proliferation. H_2_O_2_ reversibly oxidizes cysteine thiol groups of phosphatases such as PTEN, PTP-1B, and protein phosphatase 2 (PP2A) which cause loss of their activity and promote the activation of the PI3K/AKT/mTOR survival pathway [102]. ROS-dependent oxidation of v-Src causes enhancement in the invasion potential and anchorage of Src-transformed cells. ROS has been reported to confer anoikis resistance to cancer cells through the oxidation and activation of Src, leading to constitutive, ligand-independent EGFR activation and prosurvival signalling. The tyrosine kinase Src also regulates NOX1-induced generation of ROS [87]. PTEN significantly influences AKT activity under glucose deprivation. When PTEN is present in lung cancer cells, AKT phosphorylation is increased after glucose deprivation. When PTEN is mutated or knocked down, AKT phosphorylation is inhibited instead [229]. Both Src and FADK 1 are initiators of focal adhesion formation in adherent cells, contributing to cell spreading, cell migration, and prevention of cell death by anoikis. For example, in prostate cancer cells, redox-regulated anoikis resistance was mediated via Src and the EGF receptor [70]. Subsequently, it resulted in a constitutive deregulation of mitogenic pathways and proliferation independent of anchorage. It further allowed cancer cells to abolish anoikis signals and escape apoptotic responses after a loss of cell-ECM contacts [84]. Both Src and FADK 1 are initiators of focal adhesion formation in adherent cells, contributing to cell spreading, cell migration, and prevention of cell death by anoikis.

Importantly AKT signalling is involved in negative regulation of Forkhead Box Protein O (FOXO). Glucose deprivation-derived ROS production induces the nucleus translocation of FOXO and thereby promotes transcriptional activity of antioxidant-related genes [230, 231]. However, AKT inhibits this process by phosphorylating the FOXO at three conserved residues and inversely translocates FOXO from nucleus to cytoplasm [232]. One of them is ROS-dependent phosphorylation of FOXO by AKT, leading to nuclear exclusion of the factor [233–235]. Inactivation and nuclear exclusion of FOXO3a can also be elicited by H_2_O_2_ through a pathway involving AKT and the longevity-related protein p66shc [233]. Besides, AKT also promotes the ubiquitination of FOXO and leads to its degradation [236]. Therefore, FOXO activity can be inhibited by H_2_O_2_ by distinct mechanisms decreasing antioxidant production among other effects. On the other hand, AKT is also involved in mTOR activation since it inhibits Tuberous Sclerosis 2 protein (TSC2) and subsequently allows Rheb-GAP to phosphorylate mTOR1. Additionally, AKT inactivates proline-rich AKT1 substrate 1 (PRAS40), which alleviates the PRAS40-mediated inhibition of mTORC1. Therefore, AKT increases oxygen consumption and ROS production under glucose deprivation via mTOR activation [237, 238] rendering cancer cells closer to the death threshold of ROS lethality [239]. In addition, AKT is activated upon ECM detachment by activation of tyrosine kinase receptors, which has been shown to inhibit cell death mainly by promoting glucose uptake and upregulating anti-apoptotic pathways such as Bcl-2 signalling [240].

It is interesting that under glucose deprivation, AKT plays antagonistic roles from AMPK in ROS-mediated cell apoptosis because of their effects on mTOR and FOXO, but also because AMPK and AKT regulate mutual phosphorylation directly or indirectly. When glucose is abundant, AMPK activity remains limited and AKT is relatively activated, promoting cancer cell growth, division, and metastasis. Under glucose deprivation when AKT predominates, the anti-apoptotic role of AKT is reversed since glucose is lacking. However, AKT activation under glucose deprivation seems to be different among cancer cells [239, 241–243]. Thus, AKT function in this context would be specific to different cancer cells and backgrounds. In fact, it has been also reported that AKT activation can protect cells under glucose deprivation [244].

#### The MAPK signalling pathway 

ROS activate components of the JNK and p38MAPK pathways that induce apoptosis [226]. H_2_O_2_ has been shown to oxidize the redox protein thioredoxin suppressing its inhibitory effect on the p38MAPK signalling cascade [245]. Studies have demonstrated that H_2_O_2_ can promote the activation of Ras and growth factor signalling which in turn inactivates PTEN signalling cascade and activates MAPK/ERK along with PI3K/AKT/mTOR pathway. It has been suggested that small increases in ROS would be expected to activate the PI3K/AKT pathway preferentially, while further increases would be expected to trigger p38MAPK-dependent apoptosis. The activation of the Ras-Erk1/E twenty-six (2-ETS), Rac1-JNK-AP, or p38 signalling pathways would be involved in MMPs H_2_O_2_-induced expression [80, 246]. Moreover, phosphorylation of Hsp27 by ROS-activated p38 MAPK induces changes in actin dynamics in vascular endothelial cells, which may contribute to facilitate the invasive processes [247, 248]. On the other hand, JNK is able to activate some members of the FOXO transcription factor family after ROS levels are increased [249], which has been shown to induce SODs and catalase gene expression [250]. FOXO transcription factors have a complementary function to that of p53 that induce the expression of sestrin 3 [251]. Interestingly, both p53 and FOXO control a distinct set of genes that are not targets of Nrf-2 activity, even though all three factors induce HO-1 expression and Fe^2+^ storage and secretion, which is known to play a role in breast cancer progression [252]. Furthermore, the redox-sensitive transcription factors NF-κB and FOXO3a have been described as regulators of MMP expression [85, 253, 254].

#### Calcium signalling

ROS also influence the activity of calcium channels; in fact, they induce the release of calcium from cellular stores with the consequent activation of kinases, such as PKC, thereby playing important roles in the proliferation of cancer cells. Lastly, the ROS/Nrf-2/Notch1 pathway was activated by mitochondrial Ca^2+^. Mitochondrial Ca^2+^ plays a critical role in tumour progression and metastasis. In fact, the expression of the mitochondrial calcium uniporter regulator 1 (MCUR1) was reported to be higher in hepatocellular carcinoma (HCC) with metastasis and associated with tumour progression. Namely, it promoted *in vitro* invasion and *in vivo* metastasis of HCC cells by the activation of EMT via SNAI1. Likewise, treatment with the mitochondrial Ca^2+^-buffering protein parvalbumin significantly inhibited ROS/Nrf-2/Notch pathway and MCUR1-induced EMT and HCC metastasis [255]. Inhibition mitochondrial Ca^2+^ uptake, Nrf-2 expression, or Notch1 activity significantly suppressed MCUR1-induced EMT of HCC cells [255] supporting that both Notch1 and Nrf-2 are needed for MCUR1-induced EMT of HCC cells.

#### The AMPK signalling pathway

Activity of AMPK was enriched in metastatic tumours compared to primary tumours. Depletion of AMPK rendered cancer cells more sensitive to metabolic and oxidative stress, leading to the impairment of breast cancer lung metastasis [256].

AMPK mediates metabolic reprogramming by promoting catabolism and suppressing anabolism [257–260]. In part, this is due to critical maintenance of PDH, the rate-limiting enzyme involved in TCA cycle, by AMPK, which favors pyruvate metabolism towards the TCA cycle [261]. AMPK could enhance mitochondrial biogenesis and OXPHOS by activating p38 MAPK/PGC-1-α pathway [262]. In particular, PGC-1-α can act as partner of p53 influencing the impact of the mutant p53 R72 variant that is associated with poor prognosis on metabolism and metastasis in breast cancer cells, where the mutant p53 enhances migration and metastasis through the ability to bind and regulate PGC-1-α, increasing mitochondrial function [263]. Under these conditions, AMPK also inhibits mTOR1 activity which leads to decreased protein synthesis and increased autophagy. Likewise, AMPK enhances the PPP and increases NADPH production by alleviating the glucose deprivation-induced NADPH depletion via decreased fatty acid synthesis and increased fatty acid oxidation [124].

Consequently, the global inhibition of protein synthesis and autophagy induction by AMPK is known to mitigate ATP reduction under ECM-detached conditions that is associated with glucose deprivation [264, 265]. Decreased anabolism reduces ROS production, while enhanced autophagy and glycolysis increases the resilience of cells to ROS. The enhancement of autophagy and OXPHOS by AMPK also contribute to balance the input and output of energy resisting against glucose deprivation-derived ROS, which in combination with and PPP modulation contribute to modulate the redox state, relieving the ROS load under glucose deficiency. Therefore, AMPK protects DTCs from both metabolic and oxidative stress-induced cell death and facilitates cancer metastasis. The role of AMPK in anoikis resistance is supported by the results of assays where the treatment with the exogenous antioxidants Trolox and NAC can rescue ATP deficiency independent of glucose uptake [123].

The effects of AMPK on cell metabolism are achieved by its downstream effectors of which FOXO activation and mTORC1 inhibition play key roles. AMPK increases activities of FOXOs by different mechanisms including gene expression enhancement by recruiting CREB-binding protein (CBP) and p300 [230, 266, 267] and post-translational modifications as phosphorylation [268–271] and acetylation [230, 272, 273]. FOXO participates in glucose metabolism by regulating phosphoenolpyruvate carboxykinase (PEPCK) that promotes gluconeogenesis and glutamine metabolism [274, 275] and G6Pase and PGC-1-α and that promotes mitochondria biogenesis and OXPHOS [231]. FOXO activation also leads to increased oxidative stress resistance by targeting the expression of SOD, catalase, and sestrins [270]. In addition, some PGC-1-α positive cells exhibit increased ROS detoxification capacities in cancers such as melanoma (Vazquez et al. 2013). Moreover, FOXO induces expression of autophagy-related genes to elevate autophagic flux and increases the production of fatty acid and amino acids ultimately converted in products consumed by mitochondrial OXPHOS [270, 276].

In contrast, the possible antimetastatic role of AMPK has been also evidenced. AMPK blockade resulted in increased cell movement in human and murine pancreatic ductal adenocarcinoma cell models [277]. Likewise, SDF-1 decreased oxidative phosphorylation and glycolytic capacity in pancreatic ductal adenocarcinoma cells, while locked myosin light chain into a phosphorylated state, decreasing F-actin polymerization and preventing cell migration. These events were accompanied by an increase in the phosphorylated and active form of AMPK and, therefore, the described effects in the cytoskeleton would be dependent upon AMPK and the CAMKK2 [277]. Additional downstream elements seem to be necessary for metastasis enhancement for some related mutations.

### The NF-kappaB signalling pathway 

The transcription factor NF-κB is considered as a redox-sensitive transcription factor. ROS can activate NF-κB through several mechanisms and, at the same time, ROS production is also regulated by NF-κB. Several NF-κB target genes are involved in the detoxification of ROS but some of them, such as NOS, have been shown to exert a pro-oxidant function [278]. These interactions highlight a complex interplay between ROS and NF-κB. By regulating gene expression, NF-kB can promote metastasis. NF-κB can modulate EMT by inducing the expression of MMPs and key cellular adhesion molecules [85, 253, 254, 279, 280]. NF-κB upregulates the expression of MMPs, urokinase-type plasminogen activator, and cytokines in highly metastatic breast cancer cell lines. Rac-induced in response to integrin-mediated cell shape changes leads to NF-κB activation and MMP-1 production [216]. NF-κB was also suggested to be involved in the increase in the migratory potential of the MCF-7 and T47D breast cancer cells mediated by Rac [220]. In addition, ROS regulate the expression of interleukin-8 (IL-8) and the cell surface protein intercellular adhesion molecule 1 (ICAM-1) through NF-κB. Both ICAM-1 and IL-8 can regulate the trans-endothelial migration of immune cells [281, 282]. On the other hand, VEGF and its receptors are known to be regulated by NF-κB, in the promotion of angiogenesis [283, 284]. In a recent study, it was demonstrated that Kras-derived mitochondrial ROS activated NF-κB through polycystin-1 (PC1) to upregulate EGFR pro-proliferative signalling [198]. Overexpression of the inhibitor of nuclear factor kappa-B kinase (IKK) in breast cancer cells with constitutive NF-κB activity resulted in reduced expression of the receptor CXCR4 *in vitro* and the corresponding loss of migration mediated by its ligand, the SDF-1α. Introduction of CXCR4 cDNA into IκB-expressing cells restored SDF-1α-mediated migration. Thus, NF-κB regulates the motility of breast cancer cells by directly upregulating the expression of CXCR4, a receptor of the SDF-1α. NF-κB subunits p65 and p50 bind directly to CXCR4 promoter and activate its transcription. Cell surface expression of CXCR4 and the SDF-1α-mediated migration were enhanced in breast cancer cells isolated from mammary fat pad xenografts compared with parental cells grown in culture. A further increase in CXCR4 cell surface expression and SDF-1α-mediated migration was observed with cancer cells that metastasized to the lungs [285].

There are numerous interactions, links, and cooperativities between the NF-κB pathway and other signalling pathways. More recently, crosstalk of NF-κB with another transcription factor involved in certain types of cancer, that is, the transcriptional regulator ERG (ERG), has been identified. Interestingly, an increased NF-κB activity was detected in ERG fusion-positive prostate cancer patients and cell lines. It was shown that increased NF-κB activity is associated with phosphorylation of p65 on Ser536 involving signalling through Toll-like receptor 4 (TLR4) [286]. ERG also appears to stimulate the SDF-1/CXCR4 axis, which contributes to metastasis [287]. Furthermore, the redox-sensitive transcription factors NF-κB and FOXO3a have been described as regulators of MMP expression [85, 253, 254].

### The Nrf-2/ARE signalling pathway 

Nrf-2 can be activated by cigarette smoke, infection, oxidative stress, or inflammation. High amount of ROS activates tyrosine kinases to dissociate Nrf-2:Keap1 complex allowing the nuclear import of Nrf-2 [288]. Moreover, the modifications of cysteine residues in Keap1 apparently alter the interaction of Keap1 with Nrf-2 and lead to its relocation to the cytoplasm, where it is subsequently degraded by the ubiquitin proteasome [289]. Therefore, under physiological conditions, Keap1 and Nrf-2 act as a cellular sensor of damage caused by free oxygen radicals, through the constant shuttling of Keap1 between the nucleus and the cytoplasm [290]. Consequently, certain ROS levels can induce the expression of multiple antioxidants and cytoprotective genes via Nrf-2 transcriptional activity.

Because it regulates a wide spectrum of antioxidants and detoxification genes, Nrf-2 is the main inducible defense against oxidative stress [291, 292] but, whether it is more readily activated by ROS than other redox-responsive transcription factors, is unclear. Nevertheless, assuming that Nrf-2-induced gene expression provides an initial means to adapt to oxidative stress, it may offer a type of “floodgate” protection in which only once the antioxidant protection elicited by the proteins whose expression is induced by Nrf-2 is overwhelmed (and the ROS concentration threshold that induces Nrf-2 transcriptional activity can vary for different tissues and experimental systems), additional antioxidant transcription factors within the network are activated. A modification of the floodgate model would include induction by Nrf-2 of Krueppel-like factor 9 (KLF9), which is a DNA-binding transcriptional regulator that downregulates the antioxidant genes TXNRD2 and PRDX6 [293, 294]. Therefore, induction of KLF9 would shut down antioxidant defenses when ROS levels exceed a certain threshold or duration. In this scenario, other members of the network would only be activated when the antioxidant capacity of Nrf-2 target genes is exceeded or when KLF9 is induced. Once Nrf-2-directed floodgate defenses have been breached, the question of whether individual antioxidant transcription factors are activated in a stratified or coordinated manner is uncertain. In this context, it should be noted that Nrf-2 regulates the expression of HSF-1 [295] whose increased levels have been reported to be associated with metastasis [296–298] and promote infiltration, metastasis and neoangigoenesis in different cancer cell lines [299, 300]. In addition, Nrf-2 activation also led to overexpression of the NF-κB p65/RelA subunit, which antagonizes Nrf-2 by inhibiting the recruitment of CBP and activating histone deacetylase 3 (HD3) [301] and that, when oxidative stress is sufficient to cause DNA damage, ensues the activation of TP53 which in turn antagonizes Nrf-2 activity, thereby heightening oxidative stress intracellular levels and facilitating apoptosis [302]; together, these findings suggest that Nrf-2 could be downregulated by oxidative stress to an extent to which it induces inflammation and pro-apoptotic signalling.

In animal models and breast cancer patients with poor prognosis, it has been shown that Nrf-2 is activated during dormancy and in recurrent tumours. Constitutive activation of Nrf-2 accelerates recurrence, while suppression of Nrf-2 impairs it [179]. On the other hand, knockdown of Nrf-2 greatly impairs migration and invasion of a variety of cell lines including malignant and non-malignant cells [303, 304]. In a study comparing normal and tumour tissues of colorectal cancer patients, Nrf-2 and Keap1 protein levels and their tumour to normal tissue ratios were correlated with the lymph node/distant metastasis status. The authors found that the Keap1 tumour to normal tissue ratios was predictive of lympho-vascular invasion, which in turn was a significant predictor of metastasis in these patients [305].

Impairment of Nrf-2/antioxidant responsive element (ARE) pathway leads to oxidative stress, inflammation, and mitochondrial dysfunction [306]. Nrf-2 has been traditionally considered as tumour suppressor because of its cytoprotective functions against oxidative stress. However, hyperactivation of the Nrf-2 pathway creates an environment that favors the survival of normal as well as malignant cells, protecting them against oxidative stress, chemotherapeutic agents, and radiotherapy and providing a survival advantage that might favor tumour progression [307–310]. In addition, Nrf-2 also controls free Fe^2+^ homeostasis since it upregulates the expression of HO-1, which generates free Fe^2+^ via the breakdown of heme molecules. Since Fe^2+^ induces a Fenton reaction to produce the free radical ^•^OH from hydrogen peroxide, the upregulation of HO-1 leads to a paradox. In addition, Nrf-2 boosts the expression of the genes encoding several components of the ferritin complex that detoxifies Fe^2+^ by converting it to Fe^3+^ and storing it [311]. Notably, high serum concentrations of ferritin have been described in several cancers with a poor prognosis [312, 313]. As Nrf-2 induces HO-1, the enzyme catabolizing heme, its accumulation in lung cancer tissue causes the stabilization of transcription regulator protein BACH1 (BACH1), a pro-metastatic transcription factor [314–316] whose degradation is triggered by heme [317]. BACH1 pro-metastatic effects are a consequence of its interaction with the ubiquitin ligase Fbxo22 [318]. Human metastatic lung cancer displays high levels of HO-1 and BACH1 supporting the pro-metastatic role of both proteins. Moreover, BACH1 transcriptional signature is associated with poor survival and metastasis in lung cancer patients [318].

Despite Nrf-2 inhibits EMT in non-transformed cell lines [212, 319, 320], overexpression of Nrf-2 in cancer cells can enhance metastasis through the process of EMT. Namely, expression of Nrf-2 has been reported to be important for the migration of normal and malignant cells since it is needed for MMP upregulation. In this sense, it has been reported that Nrf-2 downregulation correlates with reduced expression or gelatinase activity of MMP2 and MMP9 [303, 321, 322]. Moreover, it has also been reported that Nrf-2 promotes EMT by downregulation of CDH1 expression in cancer cell lines [323, 324]. Likewise, Nrf-2 silencing reduces CDH2 expression, a process which was proposed to be mediated by the downregulation of the Nrf-2 target gene *NOTCH1* [321, 325]. Inhibition of ROS production suppressed MCUR1-induced EMT of HCC cells which would also depend on Nrf-2 and Notch1 activity [326]. Thus, ROS may act as second messengers for Nrf-2 activation leading to EMT promotion. However, the mechanism by which Nrf-2 regulates these enzymes and proteins needs to be clarified. ROS/Nrf-2/Notch1 pathway was also activated by mitochondrial Ca^2+^ [326], as discussed before in the “6.3.3” section. Interestingly, cancer cells that exhibit constitutively high levels of Nrf-2 can grow in an anchorage-independent manner and have a higher metastatic capacity [327]. Cell detachment generates ROS [328] and activates autophagy [264], which could induce Nrf-2-dependent gene expression. A possibility is that this anchorage-independent growth might be regulated by Nrf-2-dependent induction of secreted phosphoprotein 1 (SPP-1, also known as osteopontin) [329]. In cells that lose ECM attachment by adduction of integrins with the toxic metabolite methylglyoxal, Nrf-2 activation induces the expression of glyoxalase I (Glx I), which metabolizes methylglyoxal and prevents anoikis [330, 331]. Moreover, in recurrent tumours, Nrf-2 signalling induces a transcriptional metabolic reprogramming to re-establish redox homeostasis and upregulate *de novo* nucleotide synthesis. The Nrf-2-driven metabolic state renders recurrent tumour cells sensitive to glutaminase inhibition, which prevents reactivation of dormant tumour cells *in vitro* [179]. In addition, mechanistic studies showed that Nrf-2 binds to the promoter region of steroid hormone receptor (ERR1) and may function as a silencer in breast cancer cells. This may enhance RhoA protein stability and lead to RhoA overexpression that promotes migration and metastasis by the RhoA/Rho-associated protein kinase (ROCK) pathway [332]. Likewise, Nrf-2 silencing suppressed stress fiber and focal adhesion formation leading to decreased cell migration and invasion of breast cancer cells by downregulating RhoA [332]. Moreover, restoration of RhoA in MCF7 and MDA-MB-231 cells induced Nrf-2 knockdown-suppressed cell growth and metastasis *in vitro*, and Nrf-2 silencing suppressed stress fiber and focal adhesion formation leading to decreased cell migration and invasion [332].

On the other hand, Nrf-2 expression in the metastatic microenvironment has also been reported to exert an antimetastatic role. In xenograft mouse models of metastasis, whole-body and myeloid-specific Nrf-2 deletion increased susceptibility to lung metastases [333, 334]. This effect could be attributed to a persistent inflammation featured by a high number of myeloid-derived suppressor cells (MDSCs) [333] combined with redox alterations in immune cells. Redox alterations were especially relevant in MDSCs since they suppressed CD8 + T cell proliferation, depending on their intracellular ROS levels [335, 336]. Intracellular ROS levels were higher in MDSCs from Nrf-2-deficient mice as compared to those from wild-type mice [333]. In addition, MDSCs derived from Nrf-2-deficient mice produced RNS and ROS that prevented CD8 + T cell antigen recognition, a tolerance mechanism known as anergy [335, 336]. Therefore, *Nrf2* deletion would lead to a metastasis-conducive microenvironment in this xenograft model of lung cancer. In contrast, in *Keap1* knockdown mice (Keap1-kd or Keap1f/f) or in wild-type mice treated with the Nrf-2 inducer bardoxolone, the number of lung metastases were reduced [291, 337, 338]. Nrf-2 activation in Keap1f/f mice limits metastasis, probably due partly to decreased ROS levels in MDSCs [333]. Consistently, deletion of *Nrf2* or *Trsp*, the gene that encodes for selenocysteine tRNA necessary for the translation of the selenocysteine-containing antioxidant proteins GPx and Thioredoxin Reductase 1 (TR1), in the myeloid lineage confirmed that the anti-metastatic activity of Nrf-2 relates to its regulation of ROS in MDSCs [334]. Furthermore, Nrf-2-dependent downregulation of IL-6 could also prevent recruitment of myeloid precursor cells to tumours [339, 340] independently of redox regulation.

Additional proteins acting upstream Nrf-2 also have been related to metastasis. These include carbonyl reductase (NADPH) 1 (CBR1), hepatitis B virus X-interacting protein (HBXIP), nestin, and karyopherin subunit alpha-6 (KPNA6). CBR1 is another important enzyme that regulates the expression of Nrf-2 during oxidative stress and helps to detoxify ROS [341]. The levels of HBXIP that can compete with Nrf-2 for binding with Keap1 protein, via its highly conserved GLNLG motif, are positively correlated with Nrf-2 expression in breast cancer cells and clinical breast cancer tissue samples [342]. KPNA6 is a protein which facilitates nuclear import and attenuates Nrf-2 signalling, clearance of Nrf-2 protein from the nucleus, and restoration of the Nrf-2 protein to basal levels [343, 344]. Knockdown of nestin, a protein that binds to Keap1, resulted in downregulation of Nrf-2 and repressed the *in vivo* development of gastric cancer metastasis arising from the injection of SGC-7901 and MKN-45 cell lines in mice. The restoration of Nrf-2 expression, or treatment with the Nrf-2 activator sulforaphane, counteracted the inhibitory effect of nestin knockdown on the proliferation, migration, invasion, and antioxidant enzyme production [345].

### The Wnt/β-catenin signalling pathway 

Wnt signalling modulates major developmental processes and is a dominant mediator of stem cell self-renewal, cell fate, and cancer stem cell biology. β-catenin resulted upregulated in HNSCC after inhibition of human CBR1, whose expression is lower in HNSCC patients with lymph node metastasis compared to those without lymph node metastasis. In addition, CBR1 inhibition was shown to increase intracellular levels of ROS. Consistently, CBR1 inhibition resulted in increased *in vitro* invasion ability of several HNSCC cell lines and in the activation of several EMT markers, such as vimentin, CDH1, or the zinc finger protein SNAI2 (SNAI2) [346], indicating that the Wnt signalling pathway could have a pro-metastatic role. On the other hand, β-catenin expression was suppressed after induction of glutathione S-transferase omega-2 (GSTO-2), an enzyme that exhibits thioltransferase activity, in a lung squamous cell carcinoma model. The overexpression of GSTO-2 correlated with a lower oxygen consumption rate and mitochondrial membrane potential in the lung squamous carcinoma cells. Moreover, GSTO-2-overexpressing cells formed smaller tumours and the incidence of liver metastasis was lower as compared to control cells in a subcutaneous xenograft lung cancer model using nude mice. Interestingly, when cells transfected with *GSTO2* were treated with a p38 inhibitor, β-catenin expression and mitochondrial membrane potential were recovered, suggesting that GSTO-2 loss contributes to lung cancer progression, modulating tumour cells metabolism via the p38/β-catenin signalling pathway [347]. In line with these observations, pharmacological inactivation of GSK-3β, a well-known negative regulator of the Wnt/β-catenin signalling pathway, promoted the migratory activity of 4T1 murine breast cancer cells, which correlated with higher levels of ROS and functional abnormalities in the mitochondrial respiratory chain complex I/III. In addition, NOX3 and NOX4 expression were upregulated in the 4T1 cells, which would further affect the generation of ROS. Furthermore, the authors found that the expression of pSer535-eIF-2B promoted the expression of NKG2-D type II integral membrane protein (NKG2-D) ligands, Mult-1 and Rae1, followed by phosphorylation of Ser9 in GSK-3β and increased generation of ROS [255]. In the same line, the treatment with SSTC3, novel small-molecule activator of CK1α that inhibits the Wnt signalling pathway, has been proved to inhibit the growth of patient-derived metastatic colorectal cancer xenografts in mice [348, 349]. Activation of Wnt/β-catenin signalling also correlated with the activation of EMT in cervical cancer cells *in vitro* [350]. Surprisingly, GPx2 was highly expressed in cervical cancer tissues compared to normal individuals and it was showed to reduce apoptotic damage by reducing hydroperoxides and promoting proliferation and metastasis [350]. On the other hand, the modulation of the Wnt/β-catenin pathway in neighboring cells, in particular immune cells, has been relevant to study its pro-metastatic role. Indeed, pharmacological inactivation of GSK-3β downregulated NKG2-D ligands H60a and Rae1 in NK cells and suppressed their cytotoxicity [255].

Importantly, several interactions with other signalling pathways have been put of manifest. For instance, PP2A has been shown to both positively and negatively regulate Wnt pathway at multiple levels [351]. Additionally, AKT could also activate β-catenin. Through a bioinformatics analysis and Western blot assays, Ma et al. [352] showed that Zi Shen Decoction (ZSD) decreased the enzymatic activity of PI3K and AKT *in vivo* and *in vitro*. The authors also found that the AKT/GSK-3β/β-catenin pathway mediated anticancer effect of ZSD in lung cancer cells. Indeed, they showed that oral administration of ZSD suppressed the LLC growth in a subcutaneous allograft model and promoted necrosis and inflammatory cell infiltration in the tumour tissues. Pharmacological attenuation of p-GSK-3β formation by inhibiting the PI3K/AKT pathway, reversed the abovementioned effects [255]. Moreover, EGFR has also been suggested to form a complex with β-catenin contributing to invasion and the promotion of metastasis [353, 354]. Since many of the abovementioned pathways are in part related to cell redox state, Wnt/beta-catenin signalling could indirectly be conditioned by or modulate it.

### The Sonic Hedgehog signalling pathway 

Aberrant activation of Sonic Hedgehog (SHH) signalling pathway by mutations within its components drives the growth of several malignant tumours [355, 356]. Different SHH ligands have been evidenced to have some effect on the metastatic potential of lung cancer cells. In this sense, it has been reported that the canonical SHH signalling pathway is activated in lung stroma by SHH ligands secreted from transformed lung epithelia [357]. Likewise, SHH signalling dysregulation has been reported to contribute to metastasis and angiogenesis in colon cancer [358], and termination of SHH signalling through glioma-associated oncogene (GLI1) inhibition resulted in the inhibition of proliferation in colon cancer [359, 360]. Moreover, smoothened homolog (SMO) and GLI1 were highly expressed in triple negative breast cancer, and their increased expression was correlated with metastasis, poor prognosis, and recurrence of triple negative breast cancer [361]. In addition, a GLI1 isoform, called truncated GLI1, has also been reported to induce the activation of genes related to proliferation, migration, and angiogenesis of breast cancer [362].

Similarly, inhibiting SHH pathway by interfering with the interaction between the already mentioned ligands and their receptors has been shown to have an antimetastatic potential. Early abrogation of the SHH pathway using an anti-SHH/Indian hedgehog protein (IHH) antibody 5E1 against Shh, the primary Hh ligand expressed in the lung of Kras^G12D/+^;Trp53^fl/fl^ autochthonous murine lung adenocarcinoma, led to significantly worse survival with increased tumour and metastatic burden, while genetic deletion of *Shh* has no effect on survival [357]. Likewise, loss of IHH, another SHH ligand, by *in vivo* CRISPR led to more aggressive tumour growth suggesting that IHH, rather than SHH, activates the pathway in stroma to drive its tumour-suppressive effects—a novel role for IHH in the lung. Interestingly, the authors found that genetically engineered mice treated with the 5E1 antibody against SHH/IHH presented decreased blood vessel density and increased DNA damage, all of which indicated a raise in ROS and subsequent tissue damage. Indeed, Kras^G12D/+^;Trp53^fl/fl^ mice treated with the 5E1 antibody and the antioxidant NAC showed inhibited tumour growth and prolonged mouse survival, suggesting that IHH may supress tumour growth through Hh signalling pathway by quenching or detoxifying ROS through a yet to explore mechanism [357].

Hh signalling has also been shown to promote tumour metastasis by being actively involved in EMT. SHH exerts its effects on EMT via the upregulation of the SNAI1 and downregulation of CDH1 [363, 364]. Namely, ectopic expression of GLI led to increased invasiveness of pancreas cancer cell lines [365]. Likewise, metastasis was induced by overexpression of GLI1 in the rarely metastasizing clone of prostate cancer AT2.1 [364]. The role of SHH pathway in EMT was supported by the reduction in the invasive properties of pancreatic cancer cells, following SNAI1 expression downregulation [365]. Moreover, in breast cancer, SHH signalling has been shown to induce angiogenesis independently of VEGF activation [366].

## Concluding remarks

Redox disbalance is a well-known feature of tumours. The involvement of free radicals in tumour cell signalling and cancer progression is evident from the data summarized and discussed in previous sections of this review, although most studies have focused on the presence/absence of free radicals and its impact on downstream signalling at defined steps of the metastatic cascade. To better understand the impact of redox signalling in tumour cell biology and disease progression, a wider picture is needed.

A higher but sub-lethal amount of free radicals has been observed in tumour cells actively proliferating, migrating, and invading the microenvironment since free radicals, mainly ROS, are known to act as second messengers that positively modulate the signalling pathways activating these biological processes. On the other hand, tumour cells have been shown to be highly dependent on efficient detoxification of free radicals through conserved mechanisms, such as autophagy, in other phases of the metastatic cascade, namely tumour cell dissemination and dormancy. Therefore, the question might not be whether free radicals do or do not promote tumour progression; rather, the molecular and cellular context along the metastatic cascade may be determinant in the matter. Indeed, the redox status of the cell is physiologically modulated through the interplay between the pro-oxidant and anti-oxidant systems to accommodate to the changing tissue microenvironment and different stages of cell development. The extensive, but not yet conclusive, scientific data on tumour cell redox biology suggest that cancer cells highjack the molecular processes conferring physiological cellular tolerance to disbalances in redox homeostasis as well as those that favor a motile phenotype. Metastasis competent tumour cells might benefit from the cellular plasticity endowed by the activation of redox signalling pathways throughout the metastatic cascade.

These considerations may have a clinical impact when applying redox-based therapies against cancer. Indeed, current and novel chemotherapeutic approaches against cancer are already known to modulate oxidative stress. Most chemotherapeutic agents generate ROS and are known to alter redox balance in tumour cells. In this sense, anthracyclines, alkylating agents, platinum coordination complexes, and camptothecins are widely used chemotherapeutic drugs that rise ROS levels in tumour cells [367, 368]. For example, cisplatin induces ROS through mitochondrial DNA damage, which impairs the synthesis of proteins involved in the electron transport chain [369]. Other chemotherapeutic drugs like paclitaxel and doxorubicin have been shown to promote oxidative stress in cancer cells contributing, at least in part, to tumour shrinkage due to tumour cell death [370, 371]. Paclitaxel treatment showed an increase in superoxide, H_2_O_2_, and nitric oxide, as well as oxidative DNA adducts, G2-M arrest, and cells with fragmented nuclei, suggesting the involvement of ROS and RNS in paclitaxel cytotoxicity. In breast cancer, a proton pump inhibitor known as lansoprazole has been observed to increase ROS generation and supress tumour invasion. Treatment with NAC and diphenyleneiodonium, a specific inhibitor of NOX, significantly reduced lansoprazole-induced ROS accumulation [372]. In melanoma cells, the isoquinoline alkaloid berberine stimulates ROS production which in turn regulates AMPK phosphorylation and activation leading to the decrease of ERK activity and COX-2 expression, finally reducing metastatic capacity of the cells [373]. Imexon is a small prooxidant molecule that bind to cellular thiols and depletes the cysteine and glutathione stores, therefore increasing intracellular ROS. Imexon has been shown to effectively increase non-Hodgkin lymphoma cells to oxidative stress and demonstrated therapeutic benefit in a clinical trial [374]. In this sense, buthionine sulphoximine (BSO), an inhibitor of the glutamylcysteine synthetase, has been observed to also contribute to depletion of cysteine and glutathione achieving antitumour activity in several types of cancer cells [375]. The therapeutic implications of ketogenic diets through the induction of oxidative stress and in the context of its possible role in the enhancement of radio-chemotherapy responses in lung cancer xenografts have also been studied [376]. This type of diet increases dietary fatty acids, mainly PUFA levels, whose incorporation into membrane phospholipids increase cancer cells susceptibility to the accumulation of lipid peroxides and subsequent ferroptosis [377]. Moreover, the inhibition of the activity of enhanced antioxidant system in cancer cells may induce intracellular oxidative stress, for example, with the employment of drugs that block the proteasome or cause ER stress [378].

On the other side, tumour treatment through antioxidant-based therapies has also been investigated. In testicular cancer, an antioxidant cocktail of α-tocopherol, l-ascorbic acid, zinc, and selenium was used to modulate the expression of metastasis-associated protein 1 (MTA1), a gene involved in tumour growth and metastasis. The antioxidant cocktail effectively inhibited the expression of MTA1 and increased the susceptibility of tumour cells to apoptosis, suggesting that antioxidants may be helpful for metastasis prevention [379]. Furthermore, many clinical trials have been developed in order to evaluate the potential of dietary supplementation with antioxidants as suppressors of tumour development [380]. However, the context-dependent nature of redox signalling in cancer progression may contribute to the frequent contradictory results obtained in clinical trials evaluating the effect of adjuvant antioxidant therapies, in which the stage of the disease at which antioxidants are administered might influence the clinical outcome of the intervention. Therefore, much of the knowledge that we have acquired through research focused on redox signalling in cancer points to a stage-tailored strategy to develop redox-based therapies against cancer, conferring a “temporal dimension” to precision medicine.
